# White matter tract microstructure, macrostructure, and associated cortical gray matter morphology across the lifespan

**DOI:** 10.1162/imag_a_00050

**Published:** 2023-12-18

**Authors:** Kurt G. Schilling, Jordan A. Chad, Maxime Chamberland, Victor Nozais, Francois Rheault, Derek Archer, Muwei Li, Yurui Gao, Leon Cai, Flavio Del’Acqua, Allen Newton, Daniel Moyer, John C. Gore, Catherine Lebel, Bennett A. Landman

**Affiliations:** Department of Radiology & Radiological Sciences, Vanderbilt University Medical Center, Nashville, TN, United States; Vanderbilt University Institute of Imaging Science, Vanderbilt University Medical Center, Nashville, TN, United States; Rotman Research Institute, Baycrest Academy for Research and Education, Toronto, Canada; Department of Radiology, University of Calgary, Calgary, Canada; Department of Mathematics and Computer Science, Eindhoven University of Technology, Eindhoven, The Netherlands; University of Bordeaux, CNRS, CEA, Bordeaux, France; Medical Imaging and Neuroinformatic (MINi) Lab, Department of Computer Science, University of Sherbrooke, Canada; Vanderbilt Memory & Alzheimer’s Center, Vanderbilt University Medical Center, Nashville, TN, United States; Vanderbilt Genetics Institute, Vanderbilt University Medical Center, Nashville, TN, United States; Department of Biomedical Engineering, Vanderbilt University, Nashville, TN, United States; NatbrainLab, Department of Forensics and Neurodevelopmental Sciences, King’s College London, London, United Kingdom; Department of Electrical and Computer Engineering, Vanderbilt University, Nashville, TN, United States; Department of Computer Science, Vanderbilt University, Nashville, TN, United States; Alberta Children’s Hospital Research Institute (ACHRI), Calgary, Canada; Department of Radiology, University of Calgary, Calgary, Canada

**Keywords:** white matter, microstructure, macrostructure, cortex, lifespan, tractography

## Abstract

Characterizing how, when, and where the human brain changes across the lifespan is fundamental to our understanding of developmental processes of childhood and adolescence, degenerative processes of aging, and divergence from normal patterns in disease and disorders. We aimed to provide detailed descriptions of white matter pathways across the lifespan by thoroughly characterizing white matter *microstructure*, white matter *macrostructure*, and morphology of the *cortex* associated with white matter pathways. We analyzed four large, high-quality, cross-sectional datasets comprising 2789 total imaging sessions, and participants ranging from 0 to 100 years old, using advanced tractography and diffusion modeling. We first find that all microstructural, macrostructural, and cortical features of white matter bundles show unique lifespan trajectories, with rates and timing of development and degradation that vary across pathways—describing differences between types of pathways and locations in the brain, and developmental milestones of maturation of each feature. Second, we show cross-sectional relationships between different features that may help elucidate biological differences at different stages of the lifespan. Third, we show unique trajectories of age associations across features. Finally, we find that age associations during development are strongly related to those during aging. Overall, this study reports normative data for several features of white matter pathways of the human brain that are expected to be useful for studying normal and abnormal white matter development and degeneration.

## 1 Introduction

Human brain white matter is composed of fiber pathways that connect different components of the neural system. Because fiber pathways determine the brain’s functional organization, they play a critical role in nearly every aspect of cognition and behavior. These white matter tracts undergo significant changes throughout the lifespan, and characterizing how, when, and where these changes occur is critical to our understanding of developmental processes of childhood and adolescence, degenerative processes of aging, and the divergence from normal patterns of change in disease and disorder.

### 1.1 White matter microstructure

Diffusion tensor imaging (DTI) (Basser et al., 1994; Pierpaoli et al., 1996) shows robust increases in fractional anisotropy (FA) and decreases in axial, radial, and mean diffusivities (AD, RD, MD) in childhood and adolescence (Cancelliere et al., 2013; Lebel & Beaulieu, 2011; Reynolds et al., 2019), with opposite trends in healthy aging (Fjell et al., 2008; Isaac Tseng et al., 2021; Sexton et al., 2014). Cross-sectional lifespan studies reveal complex nonlinear patterns, where white matter microstructure typically reaches maturation between the early twenties to late thirties (Lebel et al., 2012; Westlye et al., 2010; Yeatman et al., 2014). Multi-compartment models of diffusion may offer increased biological specificity. One such model, the Neurite Orientation Dispersion and Density Imaging (NODDI) (Zhang et al., 2012) model provides estimates of neurite density (intracellular volume fraction; ICVF), extracellular water diffusion (isotropic volume fraction; ISOVF), and tract orientation dispersion (OD). NODDI has been used to probe biological changes in infancy (Zhao et al., 2021), childhood (Mah et al., 2017), and aging (Cox et al., 2016), but lifespan studies (Beck et al., 2021) have been reported less due to more advanced MRI acquisition requirements and limited sample sizes.

### 1.2 White matter macrostructure

Beyond the microstructural measures provided by diffusion MRI, the *macrostructural* features of white matter pathways may play a pivotal role along the aging continuum. For example, cross-sectional studies suggest large initial increases in white matter pathway volume followed by a plateau and decreases at later ages, with rates and timing of development and degradation varying across pathways, and with some relationship between white matter macrostructure and the underlying microstructural measures (Lebel et al., 2012). Moreover, other macrostructural features—including additional measures of tract volumes, areas, and lengths—have been recently measured (Yeh, 2020), but not yet characterized across the lifespan.

### 1.3 Cortical gray matter morphometry

Cortical thickness, volume, and surface area have been well-characterized (Fischl & Dale, 2000), showing developmental and aging patterns that vary regionally in the brain (Brickman et al., 2007; Frangou et al., 2022; Roe et al., 2023; F. Wang et al., 2019; Williams et al., 2023), associations with behavior and cognition (Fjell et al., 2015; Walhovd et al., 2016), and differences in neuropsychiatric disorders (Di Biase et al., 2023; L. Wang et al., 2009). However, cortical characterizations are typically performed independently of the underlying white matter connections, and the complex relationships between white matter pathways of the brain and their associated cortical structure has been less thoroughly investigated (Cafiero et al., 2019; Jeon et al., 2015; Tamnes et al., 2010).

### 1.4 A full characterization of white matter pathways across the lifespan

Previous studies have often been limited by sample size, age range, and number of features, or did not investigate specific white matter pathways of the human brain. Because of this, a few studies have investigated potential links between the developmental processes of childhood and later degenerative processes of aging (Brickman et al., 2012; Reisberg et al., 2002; Yeatman et al., 2014). In the current study, we analyze 2789 imaging datasets on participants ranging from 0 to 100 years old to segment and quantify microstructure, macrostructure, and cortical features of 63 white matter pathways. Specifically, we address four questions: (1) How are these brain features associated with age throughout the lifespan? (2) How are different features related, and how do these relationships vary across the lifespan? (3) Are age associations of different features related to each other? and (4) Are slopes of white matter age associations in development related to those later in life? In particular, we investigate a theory of retrogenesis known as the “gain predicts loss” hypothesis that proposes that pathways with features that develop faster in childhood and adolescence correspond to those that decline faster in aging (Brickman et al., 2012; Reisberg et al., 2002; Yeatman et al., 2014).

## 2 Methods

### 2.1 Datasets

The data used in this study come from the Human Connectome Project (Essen et al., 2012), which aims to map the structural connections and circuits of the brain and their relationships to behavior by acquiring high-quality magnetic resonance images. We used diffusion MRI data from the Baby Connectome Project (Howell et al., 2019), the Human Connectome Project Development (HCP-D) study, the Human Connectome Project Young Adult (HCP-YA) study, and the Human Connectome Project Aging (HCP-A) study. Within this manuscript, we will refer to these as (capitalized) *Infant*, *Development*, *Young Adult*, and *Aging* cohorts, respectively.

The Infant cohort was composed of 259 participants and 543 imaging sessions (1-5 sessions per participant) aged between 0 and 5 years. The Development cohort was composed of 652 participants aged 5 to 21 years. The Young Adult cohort was composed of 1206 participants aged 21 to 35 years. The Aging cohort was composed of 722 participants aged 35 to 100 years. After quality assurance (removal of datasets with excessive diffusion artifacts, failure of tractography, and/or failure of FreeSurfer (Fischl, 2012) or Infant FreeSurfer (Zollei et al., 2020) on the structural images), the pooled dataset was composed of 2789 imaging sessions and spanned the ages of 1 week to 100 years old. Data are summarized in Table 1.

**Table 1.** Data used in this study come from the Human Connectome Project (Essen et al., 2012) initiatives—the Baby Connectome Project (Infant), the Human Connectome Project Development (Development) study, the Human Connectome Project Young Adult (Young Adult) study, and the Human Connectome Project Aging (Aging) study.

Cohort	Subjects	Sessions	Pass QA	Age (years)
Baby HCP	259	543	388	0.03-6.1
HCP Development	652	652	622	5.5-21.9
HCP Young Adult	1206	1206	1062	22-37
HCP Aging	722	722	717	36-100

QA: Quality Assurance check for data quality + successful structural and diffusion processes. Note that Baby HCP Cohort is a longitudinal dataset.

The diffusion MRI acquisitions were slightly different for each dataset and tailored towards the population under investigation. For Development and Aging cohorts, a multi-shell diffusion scheme was used, with b-values of 1500 and 3000 s/mm^2^, sampled with 93 and 92 directions, respectively (24 b = 0). The in-plane resolution was 1.5 mm, with a slice thickness of 1.5 mm. For the Young Adult cohort, the minimally preprocessed data (Glasser et al., 2013) from Human Connectome Projects (Q1-Q4 release, 2015) were acquired at Washington University in Saint Louis and the University of Minnesota (Van Essen et al., 2012) using a multi-shell diffusion scheme, with b-values of 1000, 2000, and 3000 s/mm^2^, sampled with 90 directions each (18 b = 0). The in-plane resolution was 1.25 mm, with a slice thickness of 1.25 mm. The Infant cohort typically used a 6-shell sampling scheme with b-values of 500, 1000, 1500, 2000, 2500, and 3000 s/mm^2^, sampled with 9, 12, 17, 24, 34, and 48 directions, respectively (14 b = 0). Depending on compliance, however, a protocol matched to the Development cohort was sometimes also used for the Infant cohort (see Howell et al. (2019) for discussion on acquisition). The in-plane resolution was 1.5 mm, with a slice thickness of 1.5 mm.

For all diffusion data, susceptibility, motion, and eddy current corrections were performed using TOPUP and EDDY algorithms from the FSL package following the minimally preprocessed HCP pipeline (Glasser et al., 2013).

Structural images with T1-weighting for all cohorts were acquired with an MPRAGE sequence, with a resolution of 0.8 mm isotropic for the Infant, Development, and Aging cohorts, and resolution of 0.7 mm isotropic for the Young Adult cohort.

### 2.2 Tractography and tract features

For every session, sets of white matter pathways were virtually dissected using the TractSeg (Wasserthal et al., 2018) automatic white matter bundle segmentation algorithm. TractSeg was based on convolutional neural networks and performed bundle-specific tractography based on a field of estimated fiber orientations (Wasserthal et al., 2018). From the TractSeg outputs, we selected 63 white matter bundles for analysis, including association, commissural, thalamic, striatal, and projection and cerebellar pathways. A list of pathways and acronyms is given in the Appendix.

**Fig. 1.**
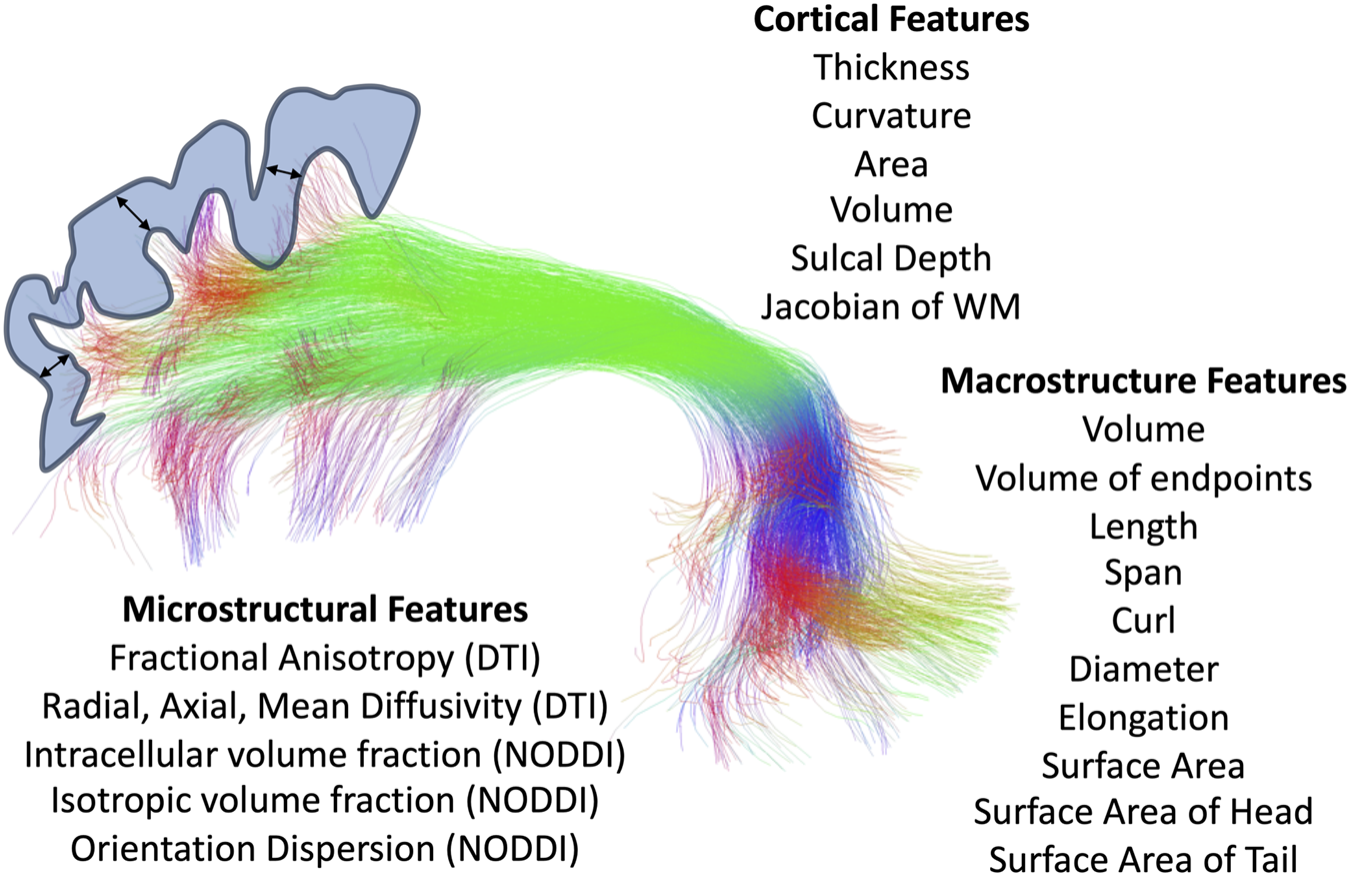
Microstructural, macrostructural, and cortical features associated with each of 63 white matter bundles.

Features of microstructure, macrostructure, and connecting cortical features were extracted for each pathway (Fig. 1). For microstructure, we first fit the DTI model to all participants using the FSL software *dtifit* algorithm (limiting fitting to b <= 1500 s/mm^2^ only), resulting in voxel-wise maps of FA, MD, AD, and RD. We then fit the NODDI model (Zhang et al., 2012) using the scilpy tractography toolbox (https://github.com/scilus/scilpy), resulting in voxel-wise maps of ICVF, ISOVF, and OD. For each tract, and each participant, these values were simply averaged across all voxels in the entire tract.

For macrostructure, we used the *scil_evaluate_bundles_individual_measures* script from the scilpy toolbox to derive 10 shape features of each tract. Briefly, these features are inspired by (Yeh, 2020), and include: tract volume (total tract volume, mm^3^); volume of endpoints (volume of voxels containing bundle endpoints, mm^3^); length (length of bundle, mm); span (distance between two ends of the bundle, mm); curl (measure of curvature ranging from 1 to infinity); diameter (bundle diameter when approximated as a cylinder); elongation (ratio of length to diameter); tract surface area (total surface area of bundle); surface area of head (surface area of beginning of bundle, i.e., the end that is most left/posterior/inferior); and surface area of tail (surface area of end of bundle, i.e., the end that is most right/anterior/superior).

Finally, cortical features associated with each bundle were computed by probing the endpoints of each streamline in a bundle (i.e., in the cortex or at the white/gray matter interface), and averaging these values across all streamlines. Cortical features were calculated from FreeSurfer run on the Development, Young Adult, and Aging cohorts, and Infant FreeSurfer run on the Infant cohort. FreeSurfer resulted in surface-based and voxel-based measures along the cortical ribbon of six cortical features: cortical thickness, volume, area, curvature (sulci have positive curvature, gyri negative, with sharper curvature indicated by higher absolute value), Jacobian of white matter (which computes how much the white matter surface needs to be distorted to register to the spherical atlas), and sulcal depth (distance in mm from mid-surface between gyri/sulci, sulci have positive values, gyri have negative values). We note that Infant FreeSurfer did not output volume nor Jacobian of white matter values. For each bundle, we used the *tckmap* feature of the MRTrix3 software package to probe the cortical thickness at the endpoints of every streamline, averaging each feature at both ends (e.g., averaging cortical thickness at the beginning and end of each streamline), and taking the average of all streamlines. This gives us features such as “the cortical thickness associated with the left Arcuate Fasciculus,” for example.

At this point, thorough manual quality assurance (QA) was performed on all datasets. Imaging sessions were removed if either diffusion or T1 data did not exist, if FreeSurfer white matter and pial surfaces or parcellation images had observable inaccuracies (frequent in the infant data due to thin, highly curved sulci, and contrast properties of T1-weighted data), excessive motion or artifacts in fractional anisotropy maps observed in diffusion data (generated through the PreQual QA pipeline (Cai et al., 2021)), or if >10% of white matter pathways (>7 pathways) were not successfully segmented (fornix and anterior commissure are frequently hard to track pathways (Wasserthal et al., 2018)).

### 2.3 Associations with age

To investigate how brain features are associated with age throughout the lifespan, we quantified the slopes of age-associations in infancy, development, young adult, and aging cohorts. For every feature of every bundle, we had data points from 2789 imaging sessions with participants ranging in age from 0 to 100 years old. When visualizing the raw data plotted against age, we noticed that the lifespan plots did not result in a smooth trajectory with age due to an offset (in nearly every feature) in the Young Adult cohort (which had different image resolution and diffusion acquisition parameters than the other three cohorts). Traditionally, data acquired from different sites and acquisitions would be harmonized, for example using the ComBat adjustment method (Fortin et al., 2017) to reduce scanner and acquisition effects. However, there is no overlap in the covariate (age) between cohorts, making this, and other, harmonization methods unfeasible. Thus, we applied a simple statistical adjustment based on continuity assumptions to harmonize data across cohorts. See Supplementary Documentation and Supplementary Figure 1 for a detailed description of this methodology.

Linear and quadratic fits are common in studies of development and aging, with quadratic fits common in lifespan studies due to the expected reversal and U-shaped curve of most features. However, quadratic fits may not be ideal because they restrict slopes on either side of the peak/minimum to be the same. Thus, less restrictive Poisson curve fits have become popular, especially in the diffusion MRI literature. Despite this, we found that Poisson fits still did not fit the highly nonlinear trends well, particularly during infancy. For this reason, all features across age were analyzed using covariate-adjusted restricted cubic spline regression (C-RCS) (Huo et al., 2016), a flexible approach to model nonlinear relationships between variables. Here, we used knots at 2, 4, 22, 35, 75, and 90 years of age, based on expected developmental shifts in volumetry (Hedman et al., 2012), with five to six knots being common as a compromise between flexibility and overfitting (Stone, 1986). The 95% confidence intervals of volumetric trajectories of each tissue/region of interest are derived by deploying C-RCS regression on 10,000 bootstrap samples.

From these C-RCS curves, the Difference per Year and Percent Difference per Year were calculated for each age as measures of cross-sectional associations with age (i.e., cross-sectional change per year or cross-sectional percent change per year). The peak/minimum of each curve can be derived, as well as the 95% confidence intervals of each peak. Results in this manuscript are displayed as curves across the lifespan and also summarized by averaging the “difference per year” value across all ages within each HCP cohort, infant (0-5), development (5-21), young adult (22-35), and aging (36+).

### 2.4 Relationships between features across a population

To examine the cross-sectional relationships between microstructure/macrostructure and cortical features, for each pathway we calculated the correlation coefficient between each feature and all other features (i.e., the correlation coefficient between 2789 x 1 vector of feature 1 and the 2789 x 1 vector of feature 2). We additionally performed partial linear correlation between all features while controlling for participant age and sex. To account for multiple comparisons, all statistical tests incorporated a false discovery rate at 0.05 to determine statistically significant relationships between features.

### 2.5 Relationships between age-associations in features

We additionally aimed to ask two questions probing the relationships between *age associations* in features. First, we asked whether greater/lesser age-associations in one feature correspond to greater/lesser age-associations in another feature. Within each cohort, for all pathways we calculated the correlation coefficients to relate the difference per year of each feature to all other features (i.e., the correlation coefficient between the 63 x 1 vector of difference per year of feature 1 for all pathways and the 63 x 1 vector of difference per year of feature 2 for all pathways).

Second, we asked whether features that develop faster in childhood and adolescence correspond to those that decline faster/slower in aging. To do this, for a given feature, we calculated the correlation coefficients to relate the cross-sectional change per year of each feature in the Development cohort to the cross-sectional change per year of the same feature in the Aging cohort (i.e., the correlation coefficient between the 63 x 1 vector of difference per year of feature 1 for all pathways in the Development cohort and the 63 x 1 vector of difference per year of the same feature 1 for all pathways in the Aging cohort). Again, statistical tests incorporated a false discovery rate at 0.05.

## 3 Results

### 3.1 How are white matter features associated with age throughout the lifespan?

#### 3.1.1 Microstructure

**Fig. 2.**
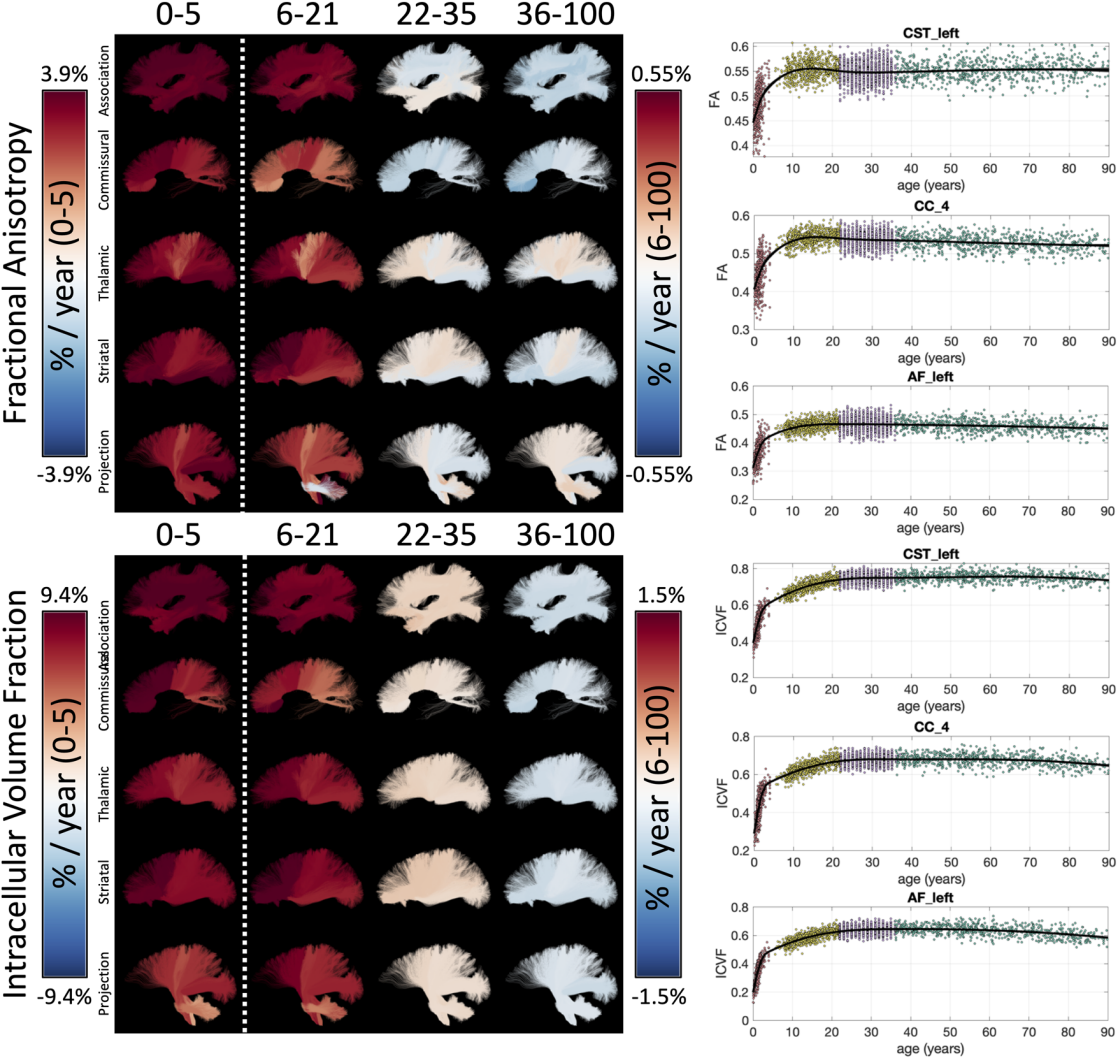
White matter microstructure shows unique lifespan trajectories. Lifespan trajectories of FA (top) and ICVF (bottom) are shown colored based on the % difference per year (cross-sectional % change per year) for each cohort (left), and data points are plotted against age for three selected pathways (right). Note 0-5 cohort is displayed on a different color scale than other cohorts.

Example lifespan trajectories of FA and ICVF are shown in Figure 2, along with bundles visualized and colored based on the % difference per year for each cohort. Age-related trends are nonlinear, yet smoothly varying, across the lifespan. In both cases, strong positive age associations in both measures are observed during infancy, continuing throughout childhood and adolescence, leveling off in young adulthood, and (typically) inverting to negative age associations in aging. Patterns of age associations also vary according to pathway location and type, with, for example, an anterior-to-posterior gradient of both measures during infancy. Other inter-pathway differences include slight negative age associations of FA in association and commissural pathways in young adulthood, and slight positive age associations in most thalamic, striatal, and projection pathways. Similar spatial patterns are observed for both FA and ICVF. Trajectories of additional microstructural features are given as Supplementary Figure 2.

**Fig. 3.**
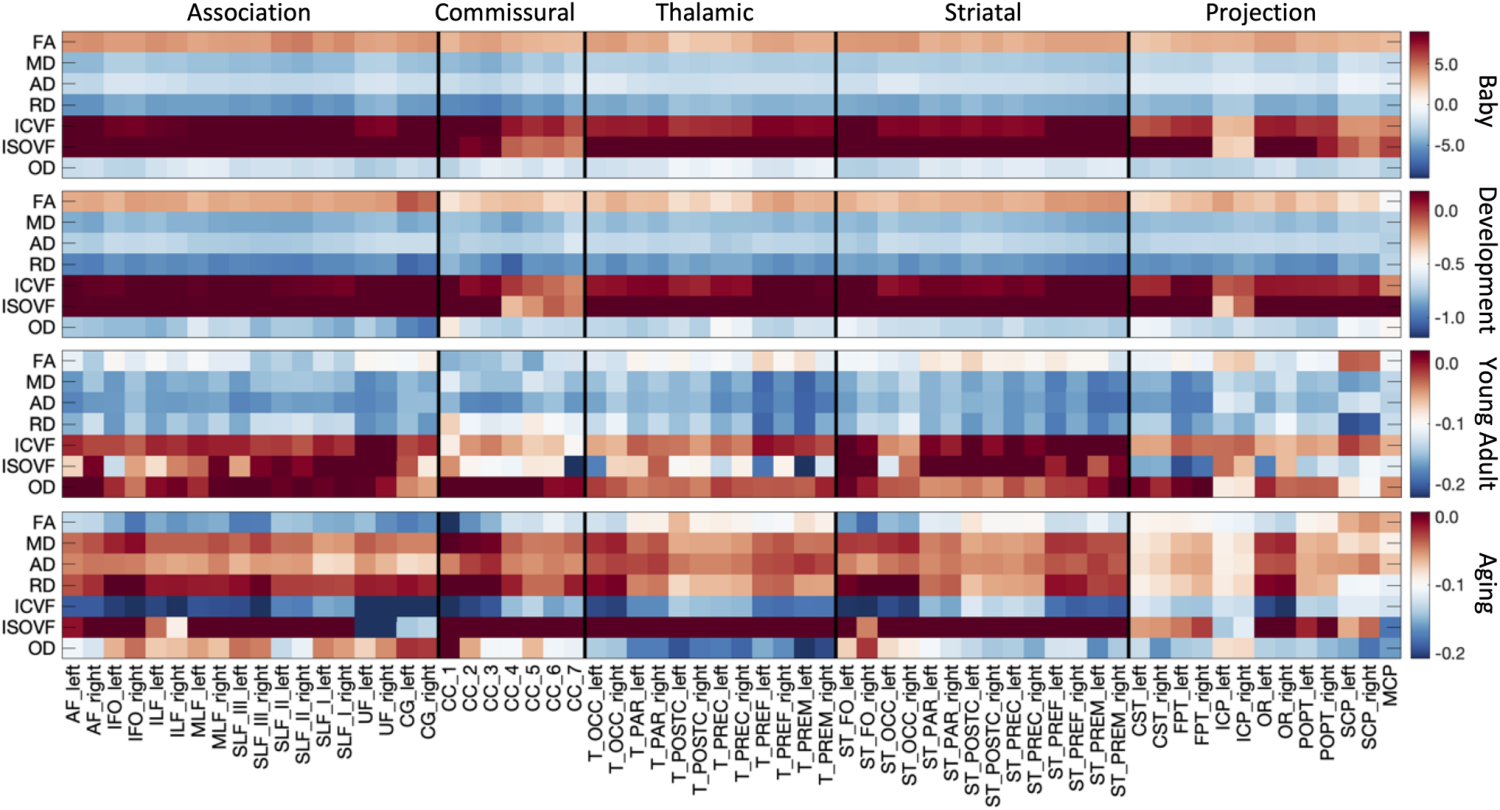
White matter microstructure shows different associations with age across pathways and across different lifespan stages. The % difference per year (cross-sectional % change per year) of all white matter microstructure features, for all bundles is shown for each of the four cohorts. Note that each cohort is visualized on a different scale to highlight trends across features.

Figure 3 summarizes the % difference per year of all white matter microstructure features, for all bundles, across all four cohorts. Here, each cohort is visualized on a different scale to highlight trends across features and pathways. In both Infant and Development cohorts, there are strong positive age associations of anisotropy, negative age associations of diffusivities (MD, AD, RD), positive age associations of ICVF, and negative age associations of dispersion. Trends are similar between these two cohorts, although age associations in Development are ~5x smaller in magnitude. Negative age associations of diffusivities continue into Young Adulthood, along with most pathways showing continued positive age associations of ICVF. However, OD now has positive age associations, while FA shows heterogenous age associations across pathways, in agreement with visualizations in Figure 2. Finally, the Aging cohort displays strong positive age associations of diffusivities, and negative age associations of anisotropy, ICVF, and OD. Notably, thalamic and projection pathways tend to display opposite patterns, with trends opposite to most association pathways for features of orientation and complexity (FA, OD), but similar trends with features of size and diffusivities (MD, AD, RD, ICVF, ISOVF).

#### 3.1.2 Macrostructure

**Fig. 4.**
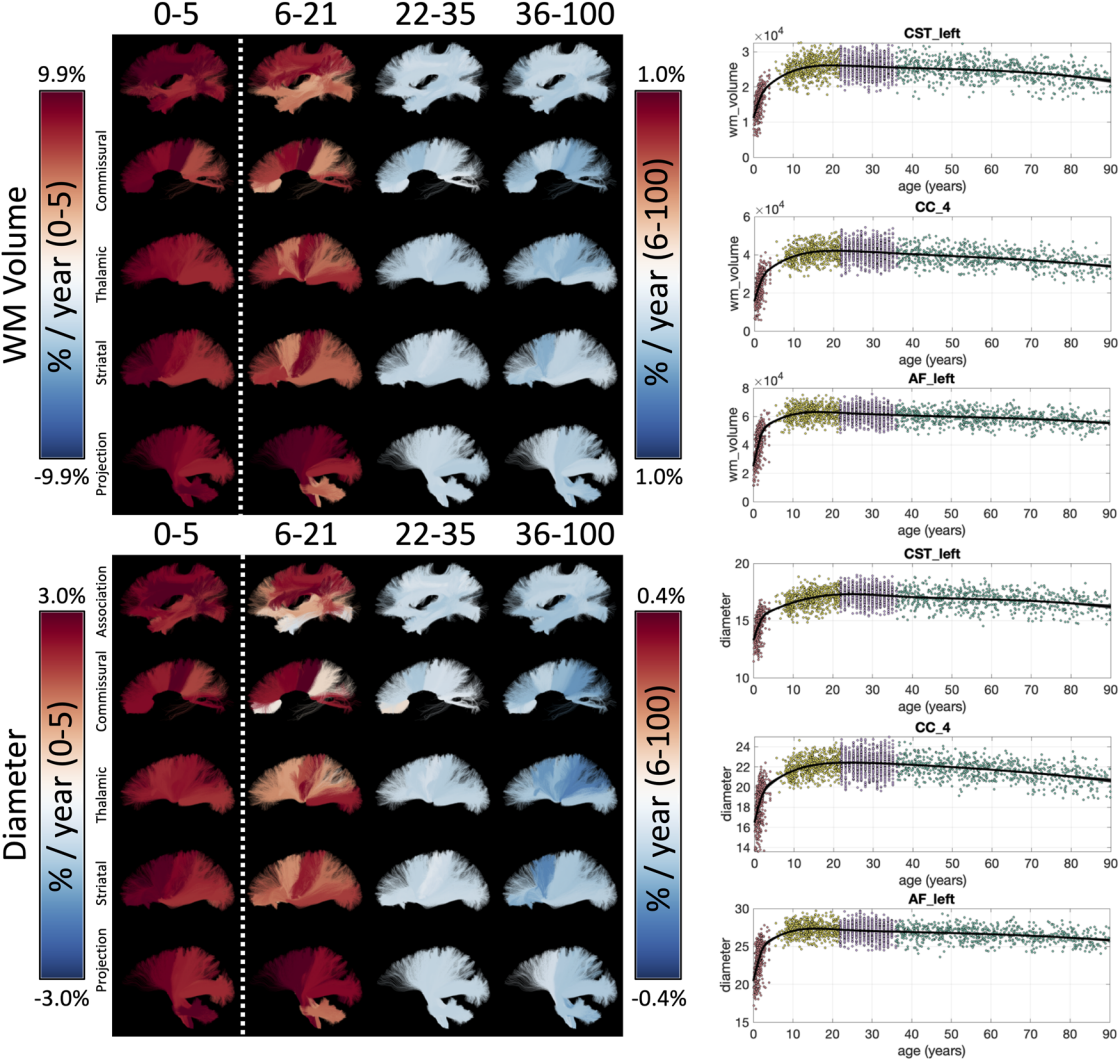
White matter macrostructure shows unique lifespan trajectories. Lifespan trajectories of WM Volume (top) and bundle diameter (bottom) are shown colored based on the % difference per year (cross-sectional % change per year) for each cohort (left), and data points are plotted against age for three selected pathways (right). Note 0-5 cohort is displayed on a different color scale than other cohorts.

Example lifespan trajectories for macrostructural features of bundle volume and bundle diameter are shown in Figure 4, again, with bundles colored based on the % difference per year. Intuitively, total volume and diameter show very similar trends across the lifespan. Again, strong positive age associations of volume occur during infancy, and continue into childhood/adolescence for most pathways, although at an order of magnitude lower % difference per year. Both features begin associating with age negatively in Young Adulthood, with the negative trends continuing into Aging, and with many bundles experiencing accelerated negative associations at older ages. Trajectories of additional macrostructural features are given in Supplementary Figure 3.

**Fig. 5.**
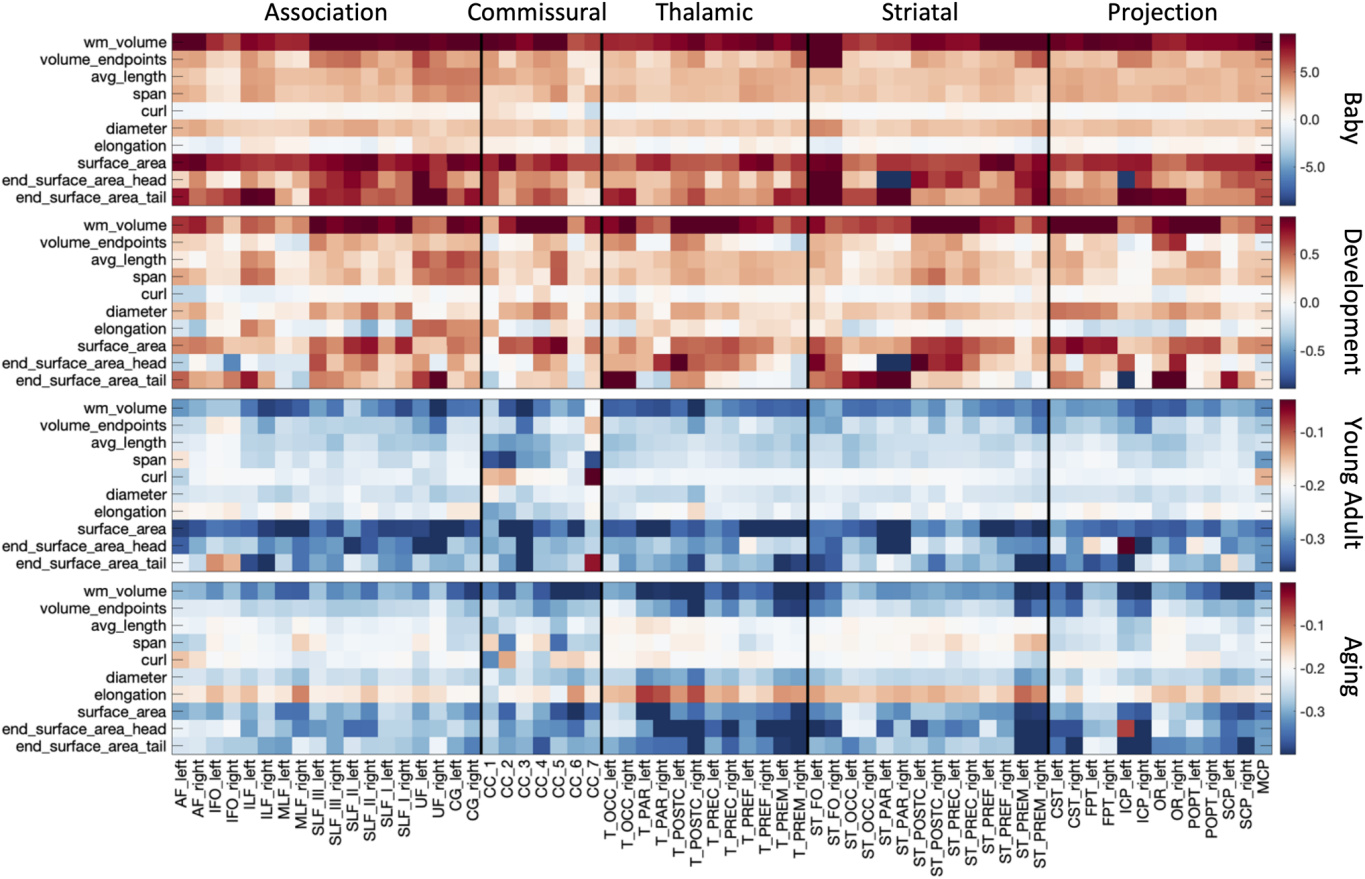
White matter macrostructure shows different associations with age across pathways and across different lifespan stages. The % difference per year (cross-sectional % change per year) of all white matter macrostructure features, for all bundles is shown for each of the four cohorts. Note that each cohort is visualized on a different scale to highlight trends across features.

The % difference per year of all white matter macrostructural features is shown in Figure 5, for all bundles, and all cohorts. Most features of volumes, length, and surface area exhibit strong positive age associations during infancy and continue in adolescence with reduced magnitudes. Pathways with connections to the pre- and post-central gyri exhibit the strongest continued positive age associations of volume and surface area into adolescence. One notable exception to the positive age associations is elongation (length to diameter ratio), possibly suggesting that the pathway width exhibits steeper positive age associations than length. A reversal of trends is clear in Young Adulthood and Aging, with nearly all features of size and shape exhibiting negative age associations in these cohorts, and with surface area generally showing the steepest negative age associations. In Aging, average length does not always exhibit negative age associations, but overall volume occupied by the pathway does exhibit negative age associations.

#### 3.1.3 Cortical structure

**Fig. 6.**
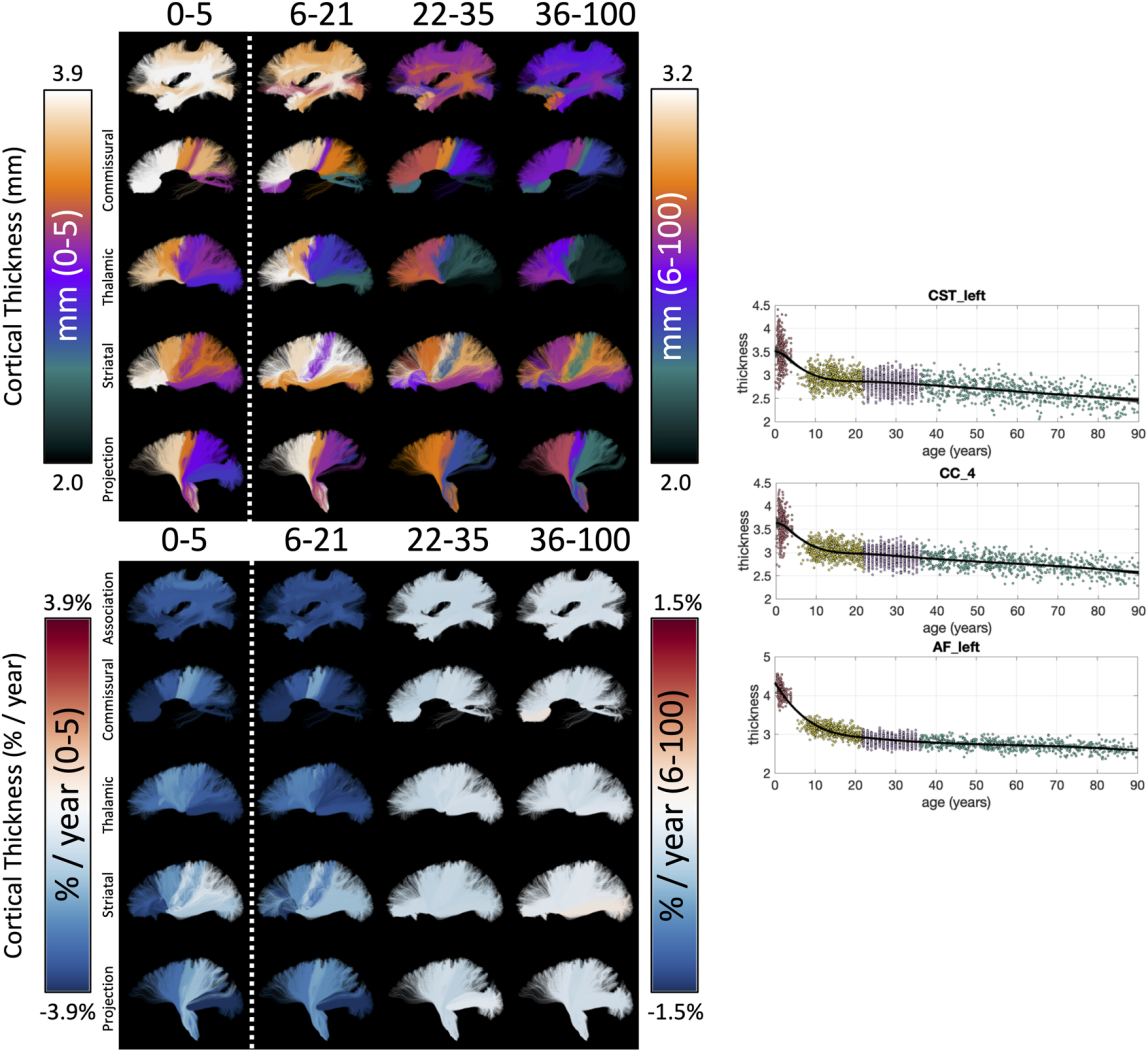
The cortical structure associated with white matter pathways shows unique lifespan trajectories. The cortical thickness of each white matter bundle at different lifespan stages is shown (top) along with the % difference per year (cross-sectional % change per year) for each cohort (bottom), and data points are plotted against age for three selected pathways (right). Note 0-5 cohort is displayed on a different color scale than other cohorts, and cortical thickness (in mm) is displayed as a different colormap (as opposed to the divergent colormap for % difference per year).

Figure 6 shows the lifespan trajectories of cortical thickness associated with each white matter bundle. Additionally, we visualize the cortical thickness associated with each bundle and the % difference per year for each cohort. First, different bundles have different associated cortical thicknesses (i.e., they connect areas with different cortical thicknesses). There is a clear pattern, with bundles connecting inferior temporal gyri, inferior frontal gyri, and superior middle frontal gyri displaying higher associated cortical thickness, and bundles connecting pre- and post-central gyri displaying lower associated cortical thickness—both in agreement with expected patterns across the cortex (Fischl & Dale, 2000; Frangou et al., 2022). Second, in our dataset (and with our CRCS fits), the cortical thickness exhibits negative age associations continually throughout the lifespan, with steepest associations in the Infant, Development, and finally the Aging datasets. Third, the difference per year varies across the pathways, with pathways connecting pre- and post-central gyri remaining relatively unassociated with age across all stages of the lifespan. Trajectories of additional cortical features are given in Supplementary Figure 4.

**Fig. 7.**
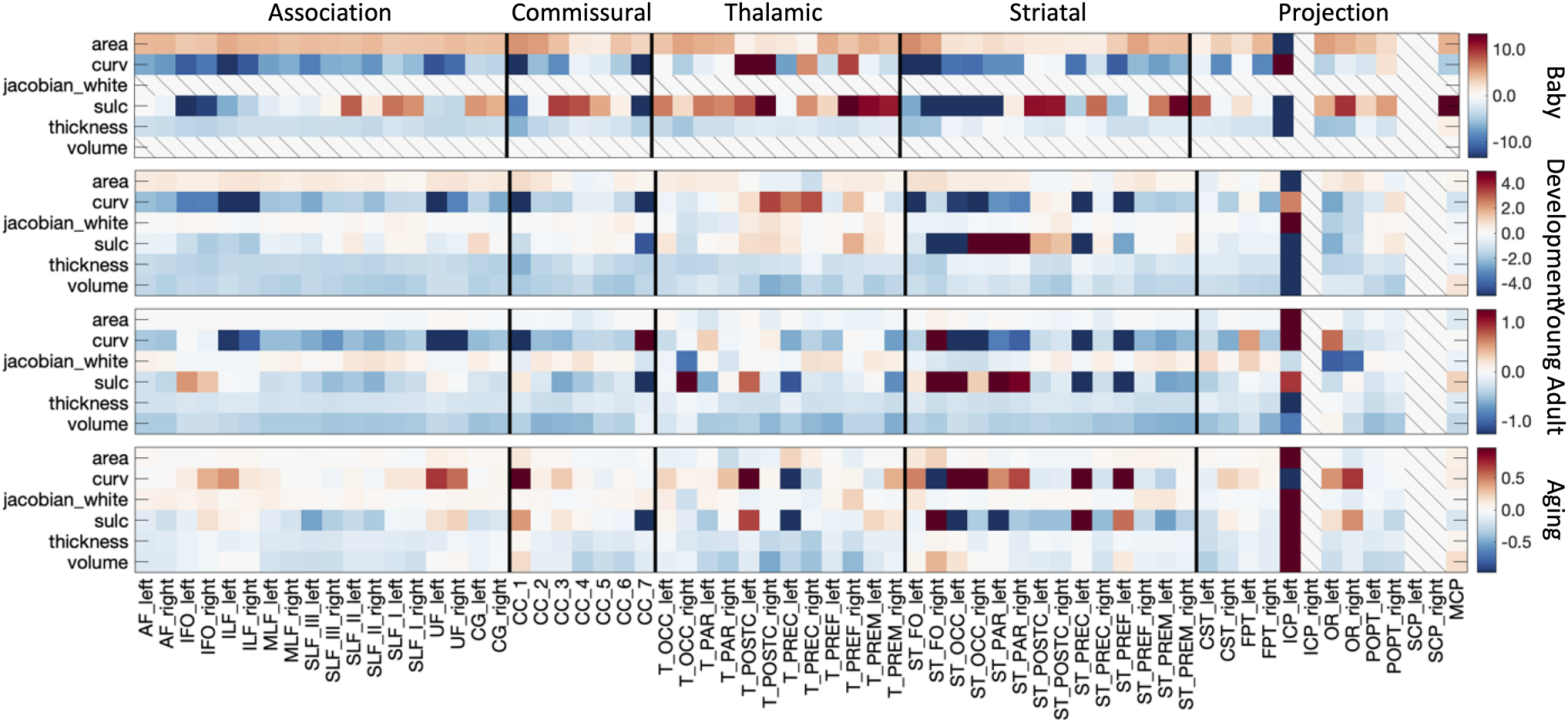
White matter cortical structure shows different associations with age across pathways and across different lifespan stages. The % difference per year (cross-sectional % change per year) of all white matter cortical features, for all bundles is shown for each of the four cohorts. Note that each cohort is visualized on a different scale to highlight trends across features. White matter volume and Jacobian are not given for Infant dataset, and several mid-brain pathways do not have associated cortex.

The % difference per year of all cortical features associated with each pathway is shown in Figure 7. The Infant cohort shows steepest positive age associations of cortical area and negative age associations of curvature and thickness for most bundles. Similarly, trends are seen in the Development cohort, with the addition of negative age associations of cortical volume (note again that neither Jacobian of white matter nor white matter volume are derived for the Infant cohort). The negative age associations of curvature, sulcal depth, thickness, and volume continue into Young Adulthood, whereas the cortical area largely plateaus for most bundles. These trends again continue into Aging.

#### 3.1.4 Spatial gradients of age-associations

**Fig. 8.**
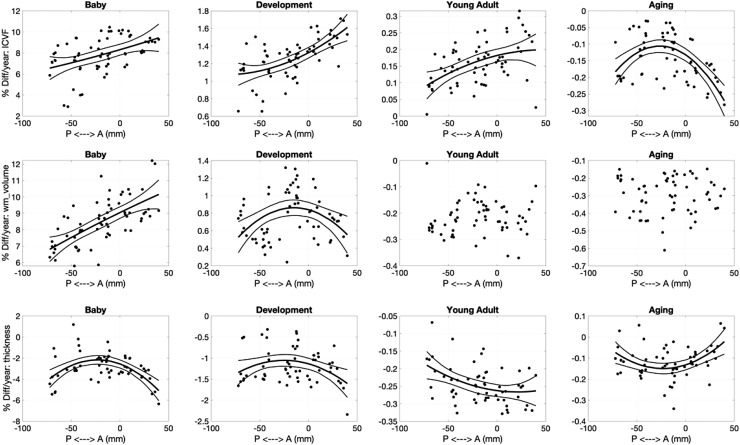
Spatial gradients in age associations across the brain exist for microstructure (ICVF; top), macrostructure (WM volume; middle), and cortical structure (thickness, bottom). Linear and quadratic trends in cross-sectional % change per year are shown across the anterior-to-posterior direction.

Beyond pathway-type differences, there is evidence for spatial gradients of age-associations across the brain. Here, we sorted pathways from anterior-to-posterior based on their average location in MNI-space and show selected results in Figure 8. Features of microstructure, macrostructure, and cortex all show significant trends that vary based on location. For example, ICVF generally shows steeper age associations for more anterior pathways in Infant, Development, and Young Adult cohorts, with a nearly reversed trend of steeper negative age associations in Aging for the most anterior and most posterior pathways. Similarly, more anterior pathways show steepest positive age associations in volume during infancy, whereas the more centralized pathways show steeper positive age associations in childhood. Finally, cortical thickness shows quadratic trends with anterior-posterior position at all stages of the lifespan, largely driven by the unique trajectories of pre- and post-central gyri and supplementary motor areas which are more centralized in the anterior-to-posterior direction.

#### 3.1.5 Relative timing of feature peak/minimum

**Fig. 9.**
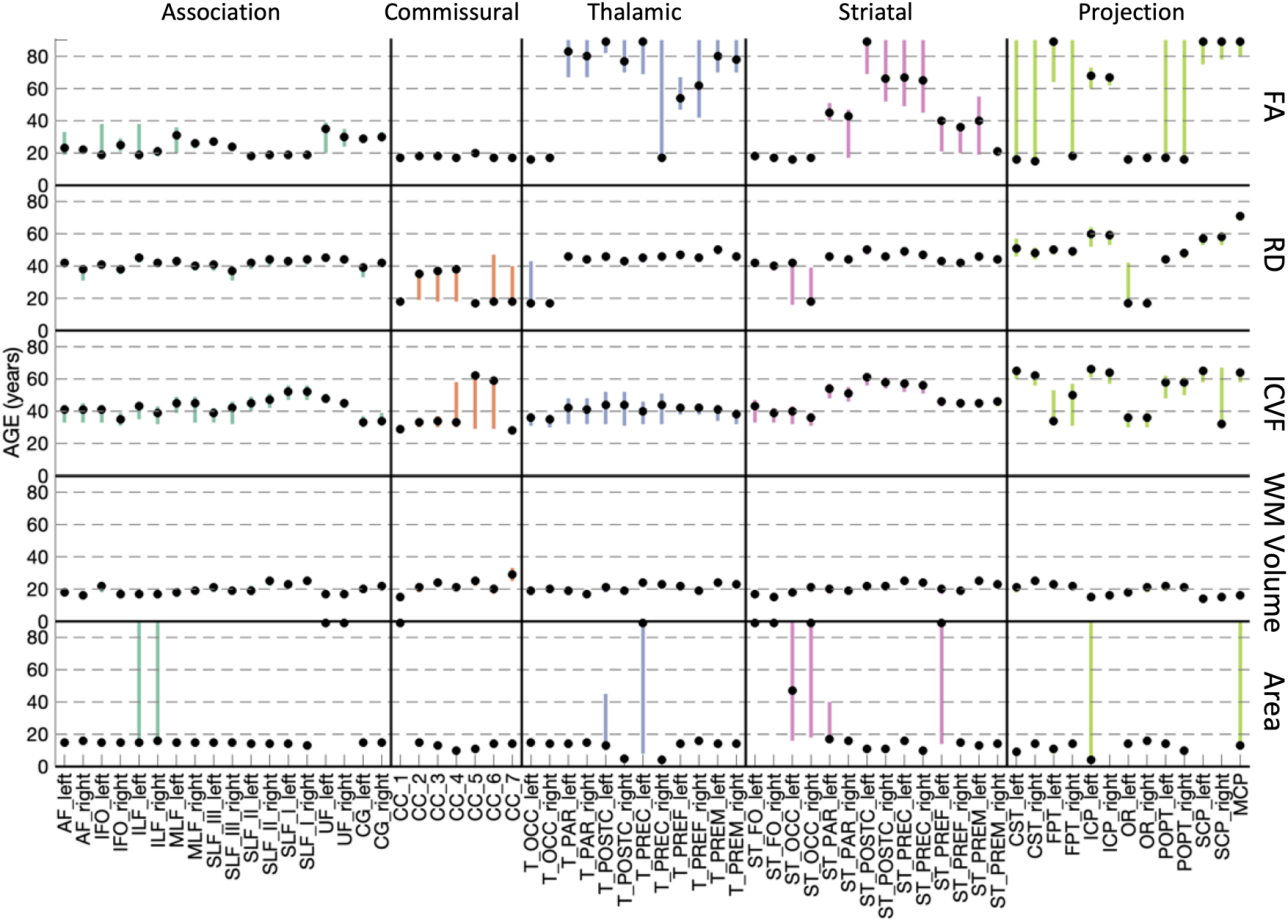
Timing of white matter feature peaks/minimum for all pathways. Marker indicates age that each pathway reaches a peak/minimum. Bars show 95% bounds of peak/minimum from bootstrapping procedures. Pathways are grouped from left to right by association, commissural, thalamic, striatal, and projection/limbic pathways.

Many features display the expected U-shaped (or inverted U-shaped) lifespan trajectory, with a peak/minimum typically occurring during young adulthood. Figure 9 shows the relative timing of white matter feature curves for selected features, indicating when the peak or minimum is reached for each pathway, along with the 95% confidence interval based on bootstrap fits. Peak anisotropy is typically reached between 20-30 years old for association pathways, just before 20 years old for commissural pathways, and with a wide variation across others—for example, many striatal, thalamic, and projection pathways show evidence of continuously positive age association of FA throughout the lifespan. ICVF and RD reverse trends (ICVF peaks, RD reaches minimum) at a later age, typically at around 40 years for most association pathways, 40-60 years for several thalamic, projection, and striatal pathways, and earlier for commissural pathways (~20 for RD, ~30 for ICVF). Pathway volume is much more homogenous, with volume of most bundles peaking within the early 20’s. Finally, cortical area associated with different pathways typically peaks before 20 years of age, although again some bundles (and associated cortical areas) do not show evidence for a single well-defined maximum throughout the lifespan.

### 3.2 How are microstructural, macrostructural, and cortical features related across the lifespan?

**Fig. 10.**
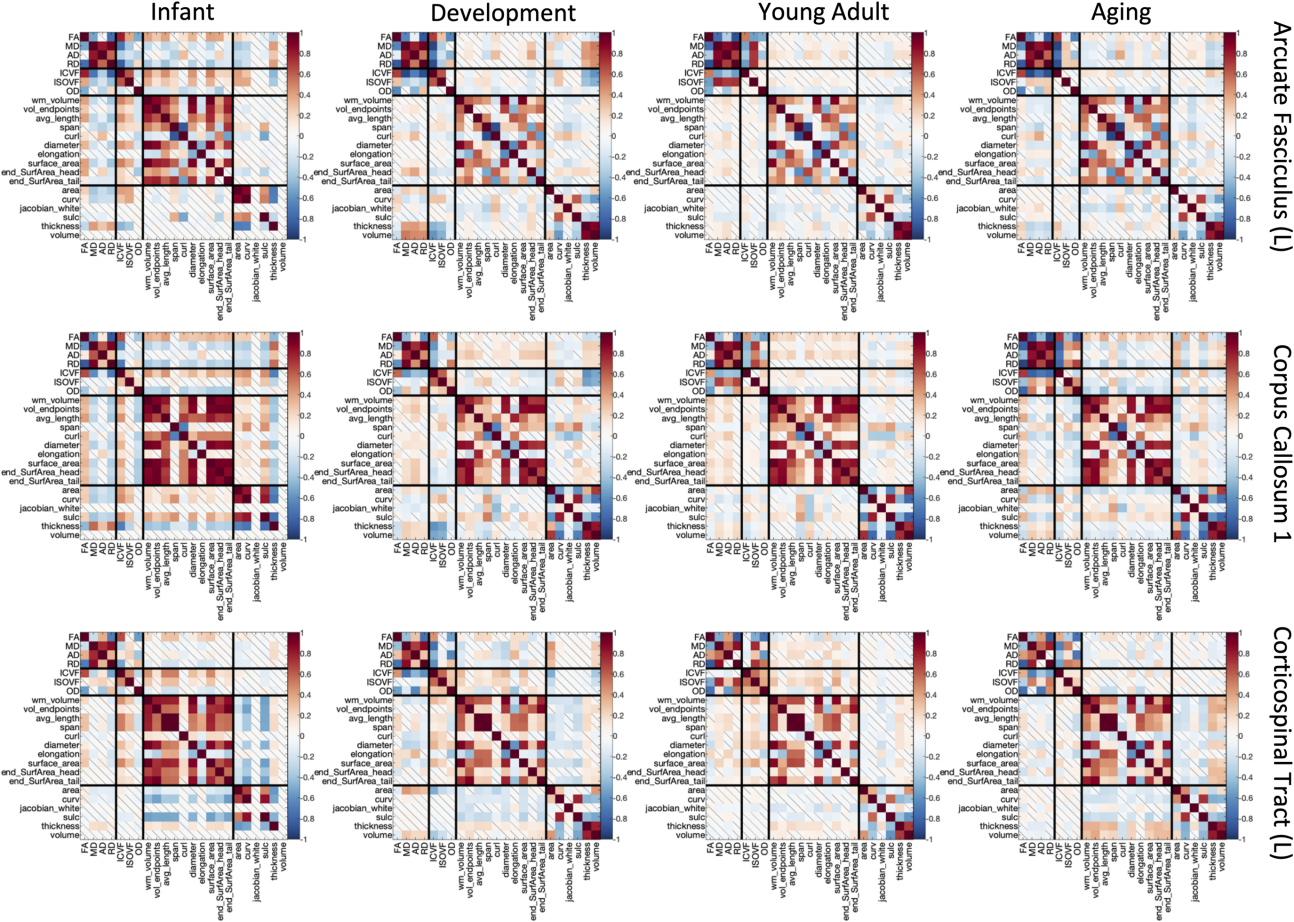
Correlations exist among white matter features of microstructure, macrostructure, and cortex, and they differ across the age cohorts and vary across pathways. Correlation coefficients are shown between all features for three selected pathways (top to bottom) and for each of four cohorts (left to right). Non-statistically significant correlation coefficients indicated by the diagonal bar in the middle of the square.

To investigate the relationship between microstructural, macrostructural, and cortical features and how these differ across the lifespan, we calculated the cross-sectional correlation of all features to each other across a population. Results for three selected pathways are shown in Figure 10. A number of observations are clear from this figure. First, regardless of cohort, microstructure measures are strongly correlated to others—diffusivities show strong positive correlations with each other (MD, AD, RD) and strong negative correlations with FA, while ICVF shows strong positive correlations with FA. Second, again intuitively, measures of volume and area generally show strong positive correlations with each other. Third, cortical thickness shows strong positive correlations with cortical volumes and negative correlations with cortical areas, sulcal depths, and curvature. Fourth are the more interesting relationships between different feature types. Some examples include the strong positive correlations between FA and features of white matter volumes, lengths, and areas across all cohorts, or the positive correlations between ICVF and those same features. Fifth, different pathways do not always show the same relationships among features. For example, the relationship between cortical thickness and microstructure does not hold true for all pathways; or the unique observation that the end beginning (head) of a bundle does not always positively correlate with features at the end (tail) of the bundle (see AF_left for an example with negative correlation in Development and Young Adulthood).

Finally, the relationship between features differs at different stages of the lifespan, as seen in Figure 10. For example, cortical thickness (highlighted with an arrow in the figure) shows negative associations with FA and positive associations with diffusivities (MD, AD, RD) during Infancy and Development, but reverses relationships within the Aging cohort. Feature relationships averaged across all pathways are shown in Supplementary Figure 6, and results for partial correlations in Supplementary Figure 7. All observations hold true, although with diminished magnitude for the partial correlations.

### 3.3 Are age associations of different features related to each other?

**Fig. 11.**
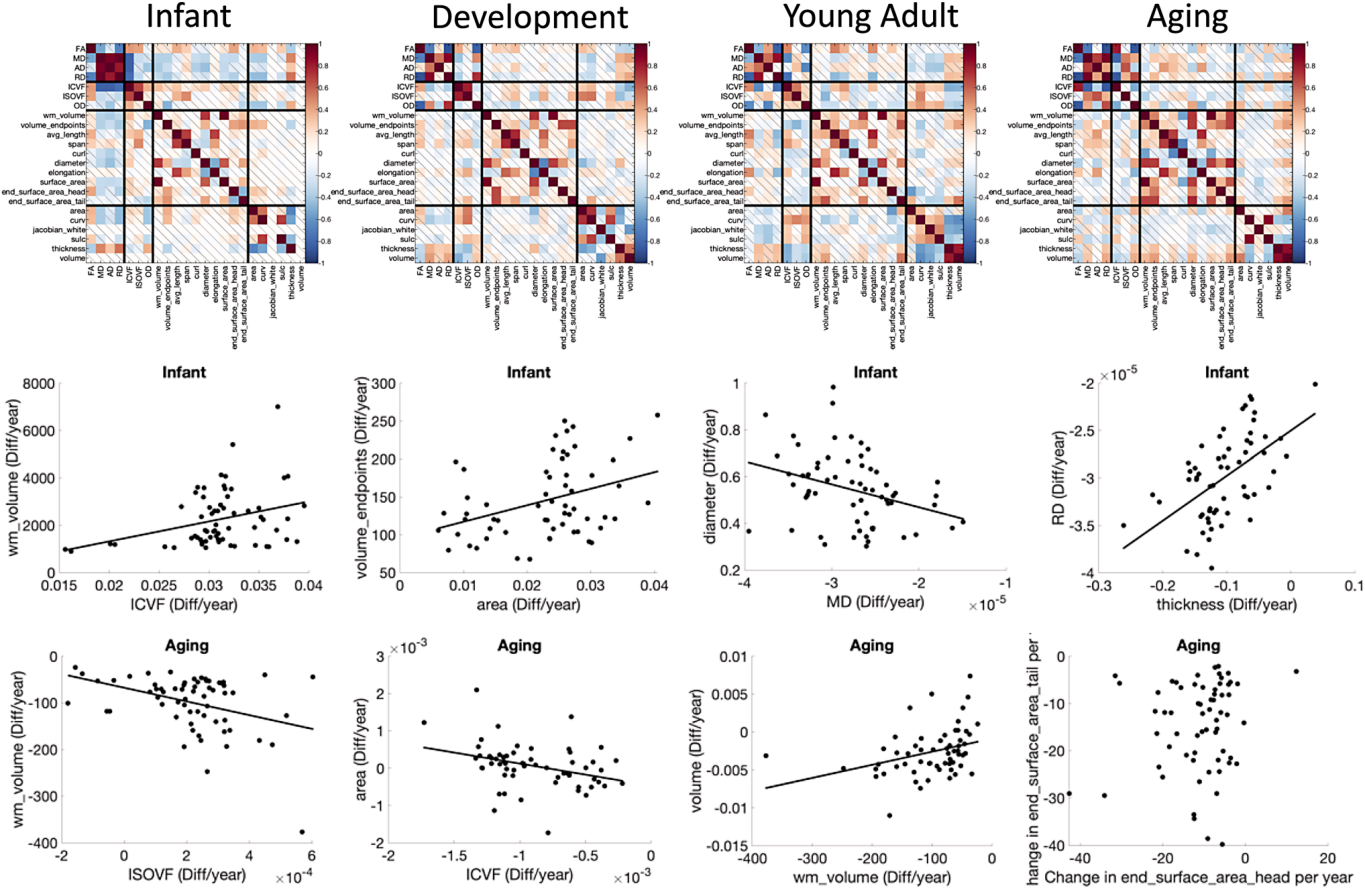
Age associations of microstructure, macrostructure, and cortical features of pathways show strong relationships. Matrices show the correlation coefficients between the difference per year (cross-sectional change per year) of features, averaged across pathways (top) and selected examples in the Infant and Aging cohorts showing the relationships between features.

We first ask, “within a cohort, do pathways with greater/smaller age-associations in one feature correspond to greater/smaller age-associations in another feature?” The correlation coefficients of the difference per year (cross-sectional rates of change) of features to each other are shown in Figure 11, with notable observations shown as additional plots. Differences in features are strongly related to each other. During infancy, pathways with steeper age-associations of white matter volume also show steeper age associations of ICVF and cortical area; pathways with steeper negative age associations of MD also show steeper positive age associations of fiber diameter; and pathways with steeper positive age associations of cortical thickness also experience steeper positive age associations of RD. These patterns also differ with age. During Aging, pathways with steeper negative age associations of white matter volume are accompanied by steeper positive age associations of ISOVF and steeper negative age associations of cortical volume; and pathways with steeper negative age associations of ICVF experience positive age associations of cortical area. Uniquely, age associations in the white matter areas at the beginning and end of pathways are not strongly correlated across pathways.

### 3.4 Does white matter pathway development influence brain degeneration later in life?

**Fig. 12.**
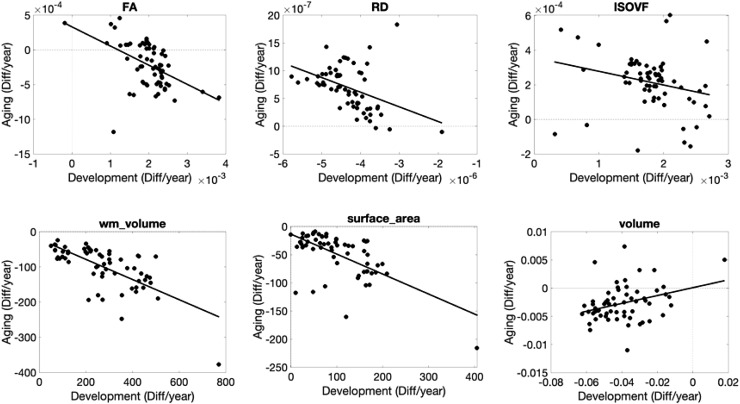
Cross-sectional change per year (difference per year) during development are strongly related to those in aging. Selected examples showing strong linear correlations are plotted, where each datapoint represents a different pathway.

Finally, we ask “do pathways with features that exhibit steeper age associations in development correspond to those that exhibit steeper age associations in aging?” Figure 12 shows several examples of features where difference per year in the Development cohort are plotted against differences per year in the Aging cohort (see Supplementary Fig. 8 for all features investigated). For microstructure—pathways that show the steepest age associations in development tend to also show the steepest opposite age associations in aging for most features. The only microstructural features that do not show statistically significant relationships between childhood and aging are AD and OD. Macrostructural features of white matter volume and surface area show similar trends, for example pathways that show the greatest age-related differences in childhood also show them in aging (i.e., CC segments, FPT, POPT, ST_PREF, and T_PREF). Finally, pathways with cortical volumes that exhibit the steepest negative age associations in development also tend to exhibit steepest negative age associations in aging, for example occipital connections (OR, T_OCC, and ST_OCC), and other striatal and thalamic pathways (T_PAR, T_PREC, ST_PREC, and ST_PAR).

## 4 Discussion

We have provided a comprehensive characterization of microstructural, macrostructural, and associated cortical features of white matter bundles across the lifespan in a large cross-sectional cohort of normal participants. We find that all features show unique lifespan trajectories, with rates and timing of development and aging that vary across pathways. Certain features tend to associate with age together, and pathway-specific trends during development bear similarities to those during aging. Characterizing the relationships among and between different features in this way may help elucidate biological changes occurring during different stages of the lifespan and make it possible to highlight atypical trajectories.

### 4.1 White matter features throughout the lifespan

#### 4.1.1 Microstructure

All *microstructure* indices demonstrated spatiotemporally varying rates of development and aging (Fig. 2). Our results are consistent with previous DTI and NODDI-based literature, but importantly link prior studies on isolated age ranges and include more white matter pathways. Throughout infancy and childhood, FA is known to increase while diffusivities (MD, AD, RD) decrease (Cancelliere et al., 2013; Lebel & Beaulieu, 2011; Lebel & Deoni, 2018; Reynolds et al., 2019), thought to reflect biological changes such as increased myelination and axonal packing (Qiu et al., 2015), with greater rates of change observed in frontal regions and limbic connections of the brain (Krogsrud et al., 2016; Reynolds et al., 2019). These changes continue into adulthood with similar regional patterns (Giorgio et al., 2010; Lebel & Beaulieu, 2011). In healthy aging, this pattern inverts, with decreases in FA and increase in diffusivities throughout much of the white matter (Barrick et al., 2010; Bennett et al., 2010; de Groot et al., 2015; Salat et al., 2005), and more pronounced changes in frontal regions (Davis et al., 2009; Isaac Tseng et al., 2021; K. Schilling et al., 2022; Sullivan et al., 2010). These changes likely reflect degradation of white matter microstructure—including demyelination, disruption of axonal structure/coherence, and increased water content. RD generally shows greater relative changes than AD in infancy and development (decreasing diffusivities) as well as aging (increasing diffusivities), driving the FA changes. Biologically, this suggests that myelination and/or packing density (increases in development, decreases in aging) may be driving these changes (i.e., RD changes (Beaulieu, 2002)) relative to differences in axonal structure and orientation coherence (i.e., AD changes (Beaulieu, 2002)).

In the current study, we found that rates of change across white matter pathways are not fully described by a simple anterior-to-posterior gradient nor by grouping connections (e.g., association, projection, or commissural pathways). For example, commissural, striatal, and thalamic pathways had stronger age associations in frontal pathways during development and aging, whereas spatial trends were not clearly visible across association and projection pathways. Further, regional variation is more pronounced in aging. For example, while FA negatively associates with older age in many pathways, some pathways remain relatively unassociated or even positively associated with old age, particularly pathways to the pre- and post-central gyri (thalamic, striatal, and corticospinal tracts) and those associated with the cerebral peduncles. These same pathways typically show negative age associations of OD—potentially reflecting either an increased coherence of neurites, or selective degeneration of cross-fibers in non-projection pathways (Han et al., 2023)—which would lead to a corresponding voxel-wise increase in FA due to a decreased partial volume fraction of cross-fibers (Wheeler-Kingshott & Cercignani, 2009).

Patterns of age associations varied across the lifespan, and there is approximately a factor of ~5 decrease in magnitude in percent change per year from infants to children, and a similar factor of ~5 decrease from children to aging. This is broadly consistent with prior studies in more isolated age ranges (Cancelliere et al., 2013; Cox et al., 2016; Lawrence et al., 2021; Zhao et al., 2021), but here we are able to combine multiple cohorts and demonstrate patterns across the entire lifespan.

#### 4.1.2 Macrostructure

All studied association, projection, thalamic, striatal, and commissural pathways showed positive age associations of volume in infancy and childhood, followed by negative age associations beginning in young adulthood that continued (often nonlinearly) during aging. These findings agree with the literature which shows large increases in volume for most tracts during development (Lebel & Beaulieu, 2011), with several pathways (CC, ILF, CST) continuing post-adolescent growth into adulthood (Lebel & Beaulieu, 2011; Lebel et al., 2012), followed by atrophy in aging (de Groot et al., 2015; Lebel et al., 2012).

Other macrostructural properties of pathways, including length, area, volume of full bundles, endpoints, and/or the trunk of bundles (Yeh, 2020), have not previously been thoroughly investigated, despite their importance for fully understanding brain development and aging. Our main finding for these other metrics, similar to volume, is that trajectories vary across pathways of the brain (Fig. 4). The volume of endpoints shows similar trends to overall volume (as above), although of smaller magnitude, meaning that the volume of the trunk decreases faster than that of endpoints. Length shows strong positive age associations during childhood, negative age associations during young adulthood, and generally negative age associations during aging, but some small positive age associations were apparent for striatal and thalamic pathways near the expanding ventricles. Tract surface area at the beginning of bundles did not always show similar age associations to those tract surface areas at the end of bundles, which suggests that the cortical areas that these connect do not atrophy at similar rates, even though they share similar connections. This might, in turn, suggest a spatial gradient driving atrophy that is not driven by structural connections. Similarly, structural covariance (Mechelli et al., 2005) across the cortex is also known to not exclusively be driven by connectivity alone (Gong et al., 2012).

#### 4.1.3 Cortical features

Here, for the first time, we directly relate specific association, projection, and commissural fiber pathways of the brain with the cortical regions that they connect, assigning cortical feature measurements to each bundle. The cortical thickness varied dramatically across bundles (Fig. 6). In general, association, commissural, and striatal pathways connecting frontal/prefrontal regions are associated with the greatest thicknesses, while those connecting with visual and motor cortices are associated with the lowest thicknesses. Second, cortical thickness of bundles are negatively associated with age across the lifespan, with their slopes varying across pathways. This is consistent with previously literature (Frangou et al., 2022).

Other features similarly offer unique insight into white matter development and interaction with the cortex. For example, much like the white matter tract surface area is positively associated with age in development and negatively associated thereafter, so is the cortical surface area, although the age associations have largely plateaued in the Aging cohort (Storsve et al., 2014). Measures of cortical volume associated with white matter pathways similarly show steep negative age associations in development (while the white matter volume associates with age positively), plateaus in young adulthood, and then continual negative age associations in aging (again as expected based on cortical studies (Sele et al., 2021; Storsve et al., 2014)). Measures of curvature and sulcal depth are less intuitive. For example, most pathways relate to the cortex with a negative curvature value (i.e., associated with gyri; see Supplementary Fig. 4), but the *age association* of curvature is positive (tends more towards 0; Fig. 7) in development and levels off with age, although it tends to remain overall negative in magnitude. While this could be interpreted biologically as morphogenesis and that pathways tend to form gyral-followed-by-sulcal connections, or that these long-range pathways primarily connect gyri (Chen et al., 2013; Nie et al., 2012), it is likely influenced by tractography biases favoring gyral connections (K. Schilling et al., 2018)—however, it could also be some combination of true favoring of gyral connections with age and greater bias in infancy/development datasets.

#### 4.1.4 Gradients across the brain

Both visually (Figs. 2, 4, and 6) and using regression (Fig. 8), we found gradients of age associations across the brain for many features. These were often quadratic, with steeper age associations observed in the most anterior and most posterior pathways. The examples in Figure 8 include ICVF, which shows a positive age association in anterior pathways during infancy, childhood, and young adulthood, and a quadratic trend in aging; the strongest age-associations were in the most anterior and posterior pathways. Total white matter volume had linear and quadratic trends in infancy and development, respectively, but no clear trends into and past adulthood. Cortical thickness showed quadratic trends at all developmental stages.

Previous studies have described spatial gradients in developmental and aging patterns. During childhood, development has been found to proceed along the posterior-to-anterior (Colby et al., 2011; Westlye et al., 2010), inferior-to-superior (Qiu et al., 2015; Storsve et al., 2016), and medial-to-lateral (Hermoye et al., 2006) axes of the brain. Similar results have been described in aging, with smaller-to-greater effects along the inferior-to-superior (Hoagey et al., 2019; Sexton et al., 2014) and posterior-to-anterior axes, and with the frontal white matter particularly vulnerable (Billiet et al., 2015; Henriques et al., 2023; Hoagey et al., 2019; Lebel et al., 2012; Sala et al., 2012; Slater et al., 2019). These observations (while not universally observed (Barrick et al., 2010; Bennett et al., 2010; Mah et al., 2017; Sullivan & Pfefferbaum, 2006)) are typically explained as a function of the later developing parts of the brain—which support higher order cognitive abilities and may be more vulnerable to age-related effects than those that support sensory or motor processes. Our results extend these observations to multi-compartment diffusion indices, macrostructure, and cortical features, and further partially explain minor discrepancies in the literature.

While most studies above describe gradients using a voxel-wise approach, we have chosen to select the center of mass of pathways in a standard space—however, pathways are not organized as parallel and perfectly ordered structures, and may in fact overlap with others within the same voxels (K.G. Schilling, Tax, Rheault, Landman, et al., 2021), and gradients as a function of position may not be fully appropriate. Regardless, these results highlight the fact that linear gradients of change may be a simplification of the development and aging process and highlight the need for pathway-specific normative trajectories across the lifespan.

### 4.2 Milestones and timings

Identifying neurodevelopmental milestones is a necessary step in characterizing normative trajectories, providing insight into biological changes in the brain, and identifying abnormal trajectories (Bethlehem et al., 2022). Here, we show that white matter volume plateaus much before FA, which, in turn, reverses trends before RD and ICVF. Bundle-specific white matter volume generally peaks around 18-25 years old, which generally occurs earlier than total white matter volume trends that reverse at ~25-28 years old (Bethlehem et al., 2022; K. G. Schilling et al., 2022). This discrepancy could be due to challenges in harmonizing lifespan curves to data from different sequences without age overlap (see *Limitations*, below), the greater flexibility in cubic splines versus linear/quadratic fitting (Lebel et al., 2012), and also the fact that we are not analyzing an exhaustive list of all brain pathways (K.G. Schilling, Tax, Rheault, Landman, et al., 2021) (e.g., the late developing short association fibers (U-fibers) which were not assessed in this work (Barkovich, 2000; Wu et al., 2014)). Second, all features show trends that are well-grouped by pathway type; for example, FA peaks for commissural fibers, association fibers, followed by the (heterogenous) peaks of striatal fibers, and finally thalamic and projection fibers. Third, these projection fibers, as well as some striatal and thalamic connections, vary widely across microstructure and cortical features, which may reflect their relative stability over the lifespan (Slater et al., 2019; Tamnes et al., 2010).

### 4.3 Feature relationships

Microstructure features were strongly related to other microstructure features. DTI is sensitive to a number of biological properties of the tissue (Beaulieu, 2002), so it is not surprising that microstructural measures were strongly correlated to one another (Chamberland et al., 2019; De Santis et al., 2014) at all points in the lifespan. Macrostructural measures also showed strong relationships to microstructure. Because volume loss, particularly in aging, may occur due to loss of myelinated fibers (Xiong & Mok, 2011), it is not surprising that volume was positively associated with ICVF, negatively associated with RD/MD, and negatively associated with ODI during aging. Finally, we found relationships between both micro/macrostructure and cortical features. As the cortex thins—likely due to loss of dendritic arbors (Nakamura et al., 1985; Scheibel et al., 1975; Vidal-Pineiro et al., 2020) in combination with reductions in synapses and shrinking of soma (Esiri, 2007) (in aging) or due to myelination (in development) (Natu et al., 2019)—the white matter also undergoes changes. For example, cortical thickness is negatively associated with MD, AD, RD, and ISOVF and positively associated with white matter volume, diameter, and endpoint volume. Finally, these associations are not the same across all pathways. While general trends described above hold true, different pathways show different associations—the process of development and aging occurs in different ways for different pathways.

Comparing rates of change between features has also been performed previously, for example finding a positive relationship between diffusivity developmental change and R1 (a relaxometry measure of myelin content) developmental change (Yeatman et al., 2014), or a negative relationship between rate of cortical thickness decrease and rate of FA increase in development (Jeon et al., 2015). These findings strengthen the hypothesis that cortical thinning is likely a result of cortical myelination (affecting the apparent white-gray matter boundary) rather than cortical pruning (Natu et al., 2019; Paquola et al., 2019; Walhovd et al., 2017), showing synchronous cortical myelination and white matter microstructural enhancement. In our study, we find similar synchronous rates of change between features during all stages of the lifespan. During infancy/development, it is intuitive that pathways with steeper age associations of macrostructure (bundle volume, bundle diameter) have corresponding steeper age associations of microstructure (ICVF, MD, respectively), or that negative age associations of cortical thickness (i.e., greater rates of myelination and/or greater pruning) are associated with negative age associations of RD (i.e., greater rates of myelination and/or greater packing density due to axon caliber enlargement (Paus, 2010)). Age associations during aging show similar intuitive trends. Pathways with the steepest positive age associations of putative interstitial spaces or diffusivities (ISOVF) experience the steepest negative age associations of cortical volume. One interesting observation is that the age associations at the beginning and end of bundles do not strongly correlate during aging, suggesting that connected cortical areas do not necessarily experience changes in the cortex (i.e., myelination and/or loss of dendritic arbors) at the same rate.

### 4.4 Development and aging relationships

For many features, a steeper slope of age associations during development corresponded to a steeper slope in aging. This finding is consistent with prior literature relating the *slopes* of age associations during development and aging (Slater et al., 2019), in contrast to the ”last in, first out” hypothesis that pathways that peak later are susceptible to earlier degeneration during aging (Brickman et al., 2012). This study provides evidence that there is a strong pathway-specific relationship between these two crucial stages of the lifespan, potentially suggesting that the rates of development of white matter feature development influence the rates of degeneration later in life.

### 4.5 Limitations

A limitation of the current study is differences in acquisition parameters and sites, which can lead to different microstructure and macrostructural measures (Fortin et al., 2017; Mirzaalian et al., 2016; Ning et al., 2020; K.G. Schilling, Tax, Rheault, Hansen, et al., 2021). The addition of more data, from different centers, and with overlaps in age/sex will ensure more appropriate harmonization and generalizability of findings.

## 5 Conclusion

In conclusion, state-of-the art tractography of 63 white matter pathways over the entire lifespan (0-100 years of age) in a large sample demonstrated age associations of many features in white matter pathways, with variation in timing and magnitude of development and aging processes. We found strong relationships between features of white matter pathways, suggesting synchronous changes in white matter microstructure, white matter macrostructure, and their connecting cortical gray matter. Finally, there are pathway-specific trends during development that are strongly related to those during aging. Together, this comprehensive characterization of white matter provides normative data that are expected to be useful for studying normal development and degeneration or compared against abnormal processes in disease or disorder.

## Supplementary Material

Supplementary Material

## Data Availability

The data used in this study come from the Human Connectome Project (Essen et al., 2012), including HCP Young Adult, HCP Aging, and HCP Development cohorts, which are freely available after appropriate data usage agreements. See the following for data download: HCP Young Adult (https://www.humanconnectome.org/study/hcp-young-adult/document/1200-subjects-data-release), HCP Aging and Development (https://nda.nih.gov/general-query.html?q=query=featured-datasets:HCP%20Aging%20and%20Development). Data derivatives from this study are packaged and ready to be shared with any HCP-authorized investigator with appropriate data usage agreement from the Human Connectome Project.

## Appendix

The bundles resulting from the TractSeg pipeline are given as a list below, with acronyms used in the text.

Association pathways: Arcuate fascicle left (AF_L); Arcuate fascicle right (AF_R); Cingulum left (CG_L); Cingulum right (CG_R); Inferior occipito-frontal fascicle left (IFO_L); Inferior occipito-frontal fascicle right (IFO_R); Inferior longitudinal fascicle left (ILF_L); Inferior longitudinal fascicle right (ILF_R); Middle longitudinal fascicle left (MLF_L); Middle longitudinal fascicle right (MLF_R); Superior longitudinal fascicle III left SLF_III_L); Superior longitudinal fascicle III right (SLF_III_R); Superior longitudinal fascicle II left (SLF_II_L); Superior longitudinal fascicle II right (SLF_II_R); Superior longitudinal fascicle I left (SLF_I_L); Superior longitudinal fascicle I right (SLF_I_R); Uncinate fascicle left (UF_L); Uncinate fascicle right (UF_R).

Commissural pathways: Rostrum (CC_1); Genu (CC_2); Rostral body (Premotor)(CC_3); Anterior midbody (Primary Motor) (CC_4); Posterior midbody (Primary Somatosensory) (CC_5); Isthmus (CC_6); Splenium (CC_7).

Projections/Cerebellar pathways: Corticospinal tract left (CST_L); Corticospinal tract right (CST_R); Fronto-pontine tract left (FPT_L); Fronto-pontine tract right (FPT_R); Inferior cerebellar peduncle left (ICP_L); Inferior cerebellar peduncle right (ICP_R); Middle cerebellar peduncle (MCP); Optic radiation left (OR_L); Optic radiation right (OR_R); Parietooccipital pontine left (POPT_L); Parieto-occipital pontine right (POPT_R); Superior cerebellar peduncle left (SCP_L); Superior cerebellar peduncle right (SCP_R).

Striatal pathways: Striato-fronto-orbital left (ST_FO_L); Striato-fronto-orbital right (ST_FO_R); Striato-occipital left (ST_OCC_L); Striato-occipital right (ST_OCC_R); Striato-parietal left (ST_PAR_L); Striato-parietal right (ST_PAR_R); Striatopostcentral left (ST_POSTC_L); Striato-postcentral right (ST_POSTC_R); Striato-precentral left (ST_PREC_L); Striato-precentral right (ST_PREC_R); Striato-prefrontal left (ST_PREF_L); Striato-prefrontal right (ST_PREF_R); Striato-premotor left (ST_PREM_L); Striato-premotor right (ST_PREM_R); Thalamo-occipital left (T_OCC_L).

Thalamic pathways: Thalamo-occipital right (T_OCC_R); Thalamo-parietal left (T_PAR_L); Thalamo-parietal right (T_PAR_R); Thalamopostcentral left (T_POSTC_L); Thalamo-postcentral right (T_POSTC_R); Thalamo-precentral left (T_PREC_L); Thalamo-precentral right (T_PREC_R); Thalamo-prefrontal left (T_PREF_L); Thalamo-prefrontal right (T_PREF_R); Thalamo-premotor left (T_PREM_L); Thalamo-premotor right (T_PREM_R).
