# Correction to: White matter tract microstructure, macrostructure, and associated cortical gray matter morphology across the lifespan

**DOI:** 10.1162/imag_x_00158

**Published:** 2024-05-20

**Authors:** Kurt G. Schilling, Jordan A. Chad, Maxime Chamberland, Victor Nozais, Francois Rheault, Derek Archer, Muwei Li, Yurui Gao, Leon Cai, Flavio Del’Acqua, Allen Newton, Daniel Moyer, John C. Gore, Catherine Lebel, Bennett A. Landman

**Affiliations:** Department of Radiology & Radiological Sciences, Vanderbilt University Medical Center, Nashville, TN, United States; Vanderbilt University Institute of Imaging Science, Vanderbilt University Medical Center, Nashville, TN, United States; Rotman Research Institute, Baycrest Academy for Research and Education, Toronto, Canada; Department of Radiology, University of Calgary, Calgary, Canada; Department of Mathematics and Computer Science, Eindhoven University of Technology, Eindhoven, The Netherlands; University of Bordeaux, CNRS, CEA, Bordeaux, France; Medical Imaging and Neuroinformatic (MINi) Lab, Department of Computer Science, University of Sherbrooke, Canada; Vanderbilt Memory & Alzheimer’s Center, Vanderbilt University Medical Center, Nashville, TN, United States; Vanderbilt Genetics Institute, Vanderbilt University Medical Center, Nashville, TN, United States; Department of Biomedical Engineering, Vanderbilt University, Nashville, TN, United States; NatbrainLab, Department of Forensics and Neurodevelopmental Sciences, King’s College London, London, United Kingdom; Department of Electrical and Computer Engineering, Vanderbilt University, Nashville, TN, United States; Department of Computer Science, Vanderbilt University, Nashville, TN, United States; Alberta Children’s Hospital Research Institute (ACHRI), Calgary, Canada; Department of Radiology, University of Calgary, Calgary, Canada

Figure 3andFigure 5have been updated due to formatting issues with the colorbar labels on several subplots. These labels have been corrected, however, the color and format of the rest of the figure remains unchanged, and no results nor interpretation are changed by the formatting issue.

**Fig. 3.**
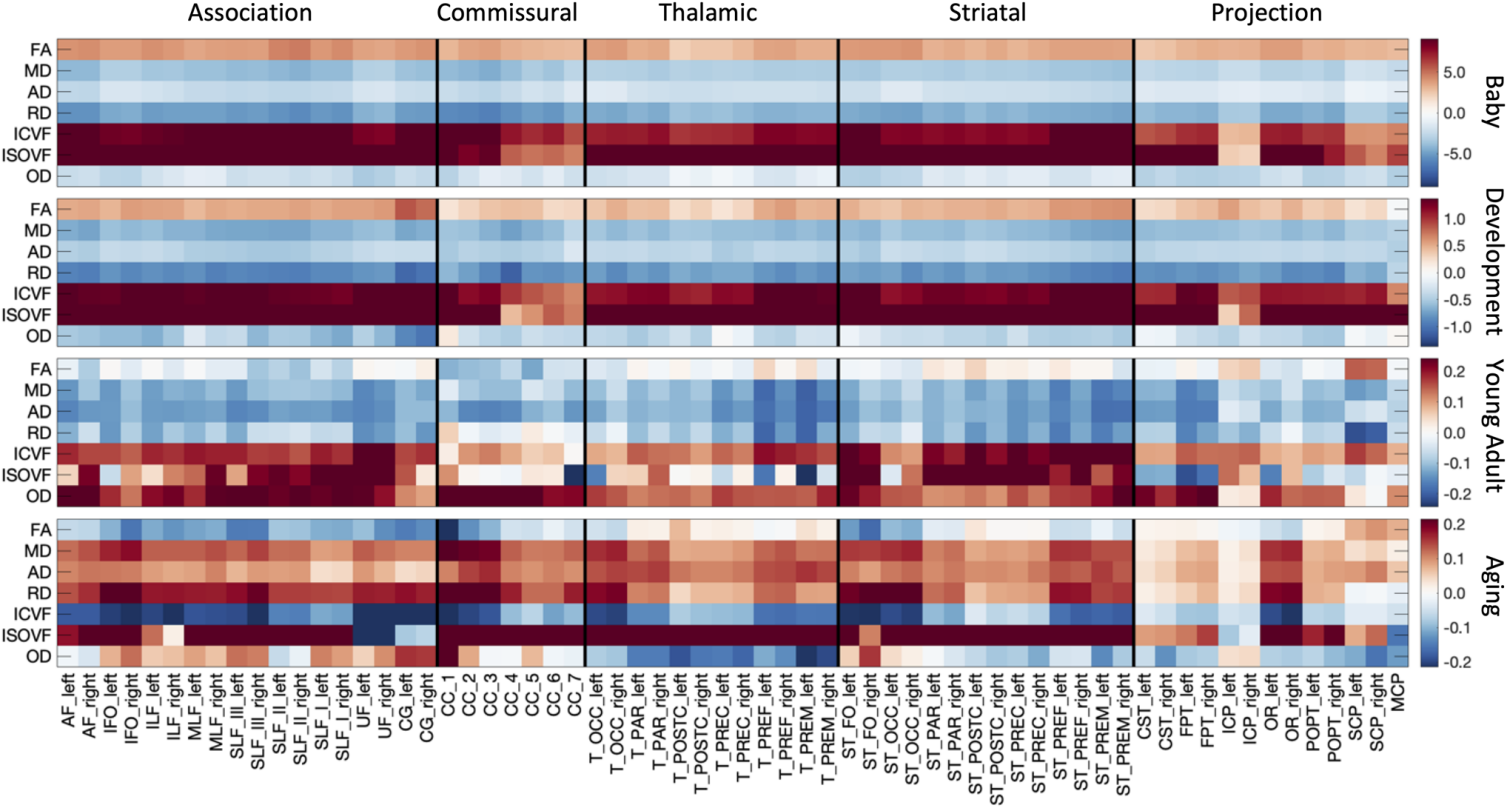
White matter microstructure shows different associations with age across pathways and across different lifespan stages. The % difference per year (cross-sectional % change per year) of all white matter microstructure features, for all bundles is shown for each of the four cohorts. Note that each cohort is visualized on a different scale to highlight trends across features.

**Fig. 5.**
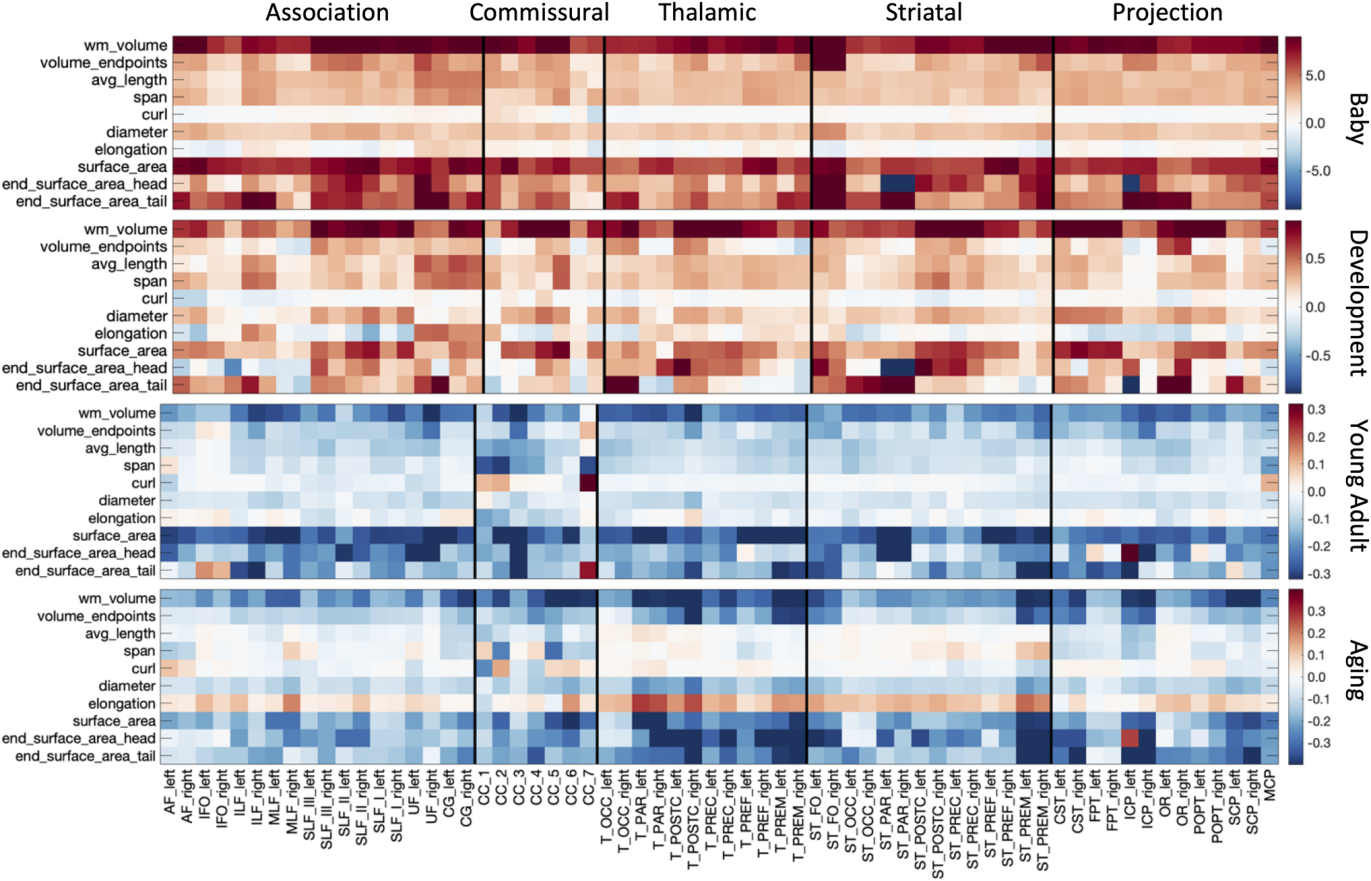
White matter macrostructure shows different associations with age across pathways and across different lifespan stages. The % difference per year (cross-sectional % change per year) of all white matter macrostructure features, for all bundles is shown for each of the four cohorts. Note that each cohort is visualized on a different scale to highlight trends across features.

