# Supplementary material for: White matter tract microstructure, macrostructure, and associated cortical gray matter morphology across the lifespan
Source: Imaging Neurosci (Camb). 2023 Dec 18;1:imag-1-00050. doi: 10.1162/imag_a_00050 (PMC12007540; doi:10.1162/imag_a_00050)
Supplement: Supplementary Material [file imag_a_00050-supp.pdf]

Supplementary Information

White matter tract microstructure, macrostructure, and associated cortical gray matter morphology across the lifespan

Kurt G Schilling[1,2], Jordan A. Chad[3,4], Maxime Chamberland[5], Victor Nozais[6], Francois Rheault[7], Derek Archer[8,9], Muwei Li[1,2], Yurui Gao[2,10], Leon Cai[10], Flavio Del'Acqua[11], Allen Newton[1,2], Daniel Moyer[12,13], John C. Gore[1,2,10], Catherine Lebel[14,15], Bennett A Landman[1,2,12,13]

1. Department of Radiology & Radiological Sciences, Vanderbilt University Medical Center, Nashville, TN, USA.
2. Vanderbilt University Institute of Imaging Science, Vanderbilt University Medical Center, Nashville, TN, USA.
3. Rotman Research Institute, Baycrest Academy for Research and Education, Toronto, ON, Canada
4. Department of Radiology, University of Calgary, Calgary, AB, Canada
5. Department of Mathematics and Computer Science, Eindhoven University of Technology, Eindhoven, The Netherlands
6. Univ. Bordeaux, CNRS, CEA, Bordeaux, France
7. Medical Imaging and Neuroinformatic (MINi) Lab, Department of Computer Science, University of Sherbrooke, Canada
8. Vanderbilt Memory & Alzheimer's Center, Vanderbilt University Medical Center, Nashville, TN, 37212, USA
9. Vanderbilt Genetics Institute, Vanderbilt University Medical Center, Nashville, TN, 37232, USA
10. Department of Biomedical Engineering, Vanderbilt University, Nashville, TN, USA
11. NatbrainLab, Department of Forensics and Neurodevelopmental Sciences, King's College London, London UK.
12. Department of Electrical and Computer Engineering, Vanderbilt University, Nashville, TN, USA
13. Department of Computer Science, Vanderbilt University, Nashville, TN, USA
14. Alberta Children's Hospital Research Institute (ACHRI), Calgary, AB, Canada
15. Department of Radiology, University of Calgary, Calgary, AB, Canada

As described in the main text - for every feature of every bundle, we had data points from 2789 imaging sessions with participants ranging in age from 0 to 100 years old. When visualizing the raw data plotted against age, we noticed that the lifespan plots did not result in a smooth trajectory with age due to an offset in the young adult cohort (which had different image resolution and diffusion acquisition parameters than the other three cohorts). Traditionally, data acquired from different sites and acquisitions would be harmonized, for example using the ComBat adjustment method [1] to reduce scanner and acquisition effects. However, there is no overlap in the covariate (age) between cohorts, making this, and other, harmonization methods unfeasible. Thus, we applied a simple statistical adjustment based on continuity assumptions to harmonize data across cohorts.

Specifically, we chose HCP Aging as the standard feature space in which to transform our data. This subset of the data was fit to linear mixed effects models with linear and quadratic trends. Bayesian Information Criterion [2] used for final model selection. Similar fitting was performed for the Young Adult dataset, which immediately proceeds the Aging dataset in age ranges. From this, a simple scaling factor was determined which ensures matching of the overlapping endpoints in the age range of each cohort. This scaling factor was applied to the feature-under-investigation for the entire Young Adult cohort. This procedure was applied sequentially, next to the Development, and finally the Infant cohorts. Example plots showing pre- and post-harmonization are shown in **Supplementary Figure 1**. In all cases, clear discontinuities are present pre-harmonization, and largely removed post-harmonization. Most post-harmonization curves are further continuous in the first order derivatives, although some nonlinear over/undershoot remains. For this reason, rates of change in the manuscript are given over entire age ranges rather than any discrete age.

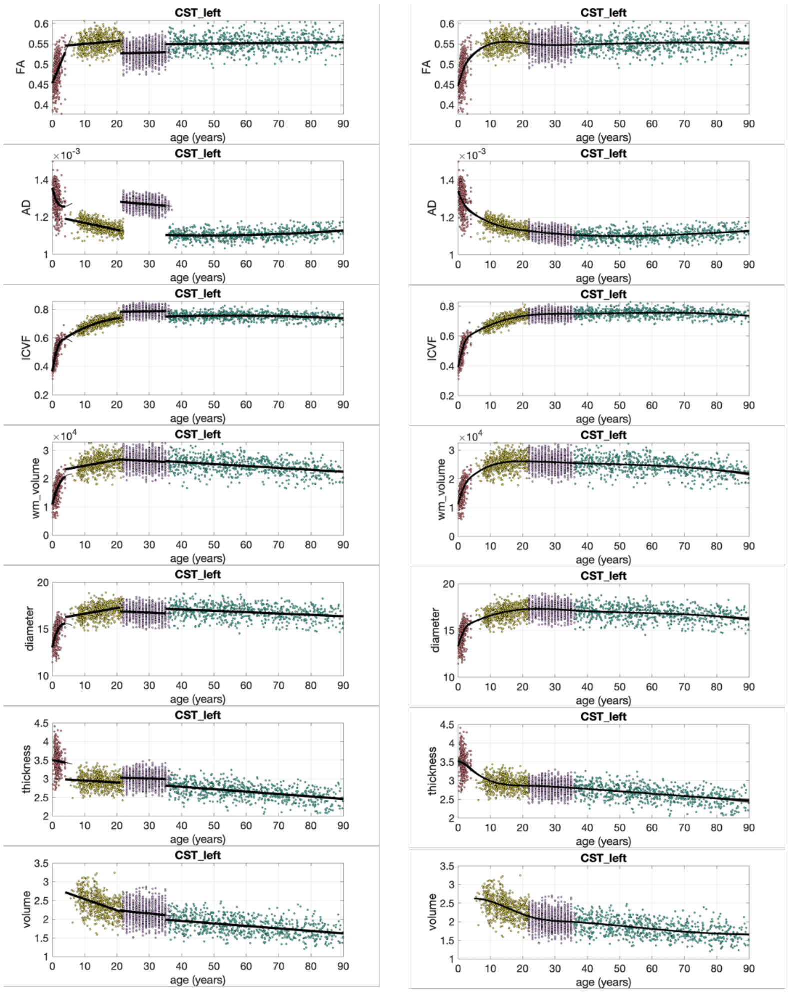

***Supplementary Figure 1**. Pre-harmonization (left) and post- harmonization (right) of example features of the cortical spinal tract (CST). Data points are plotted against age with each cohort displayed as a different color. Linear/Quadratic models (left) were used to perform statistical harmonization, resulting in a continuous curve when fitting with cubic-spline regression (right).*

Figure 2 in the main manuscript shows example lifespan trajectories of FA and ICVF. For completeness, trajectories of additional microstructural features (for AF_left, CST_left, and CC_4) are shown in **Supplementary Figure 2**.

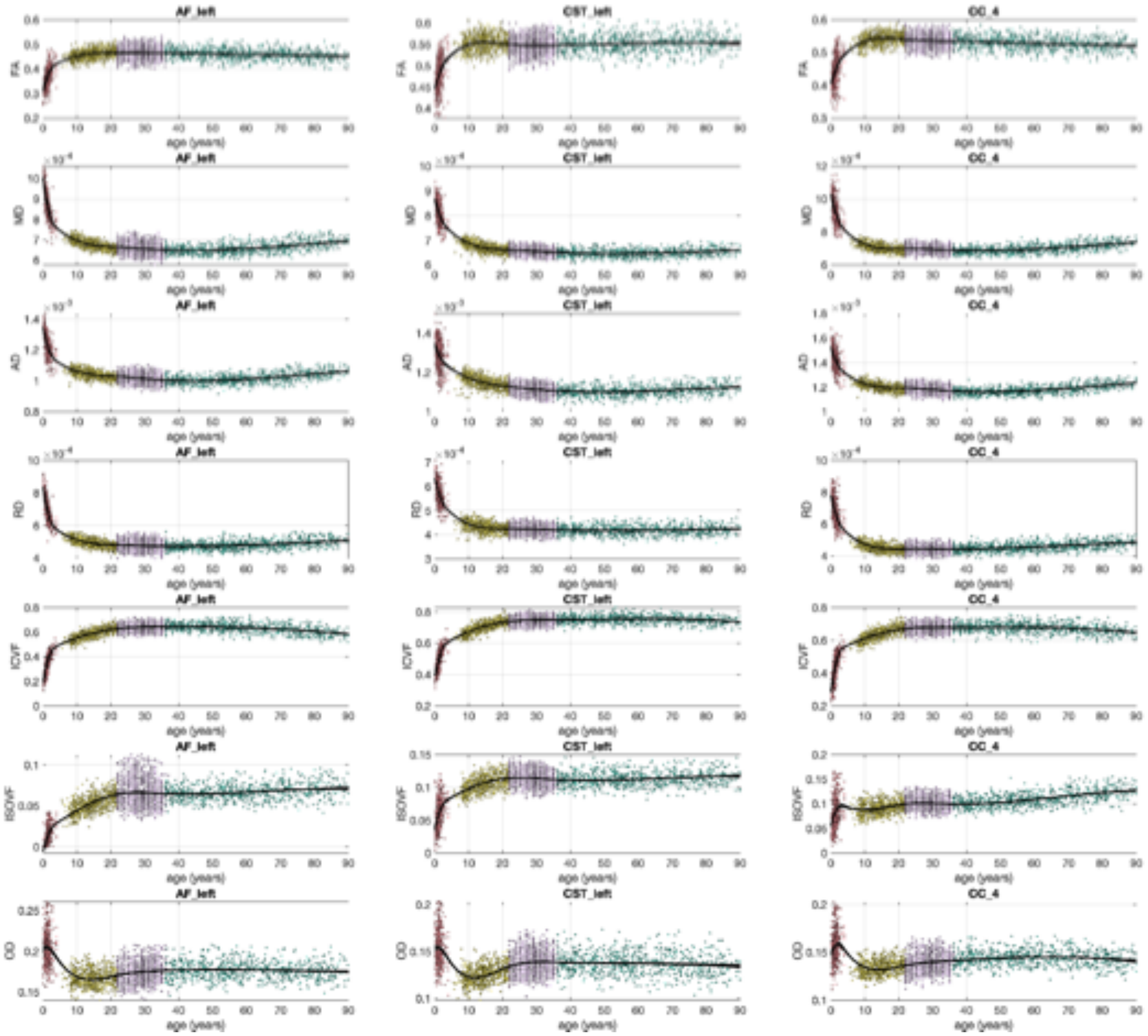

Supplementary Figure 2*. White matter microstructure shows unique lifespan trajectories. Lifespan trajectories of DTI indices (FA, MD, AD, RD) and NODDI indices (ICVF, ISOVF, OD) are plotted against age for three selected pathways.*

Example lifespan trajectories for macrostructural features of bundle volume and bundle diameter are shown in **Figure 4** of the main manuscript. For completeness, we show ten additional macrostructural features for the same pathways in **Supplementary Figure 3**.

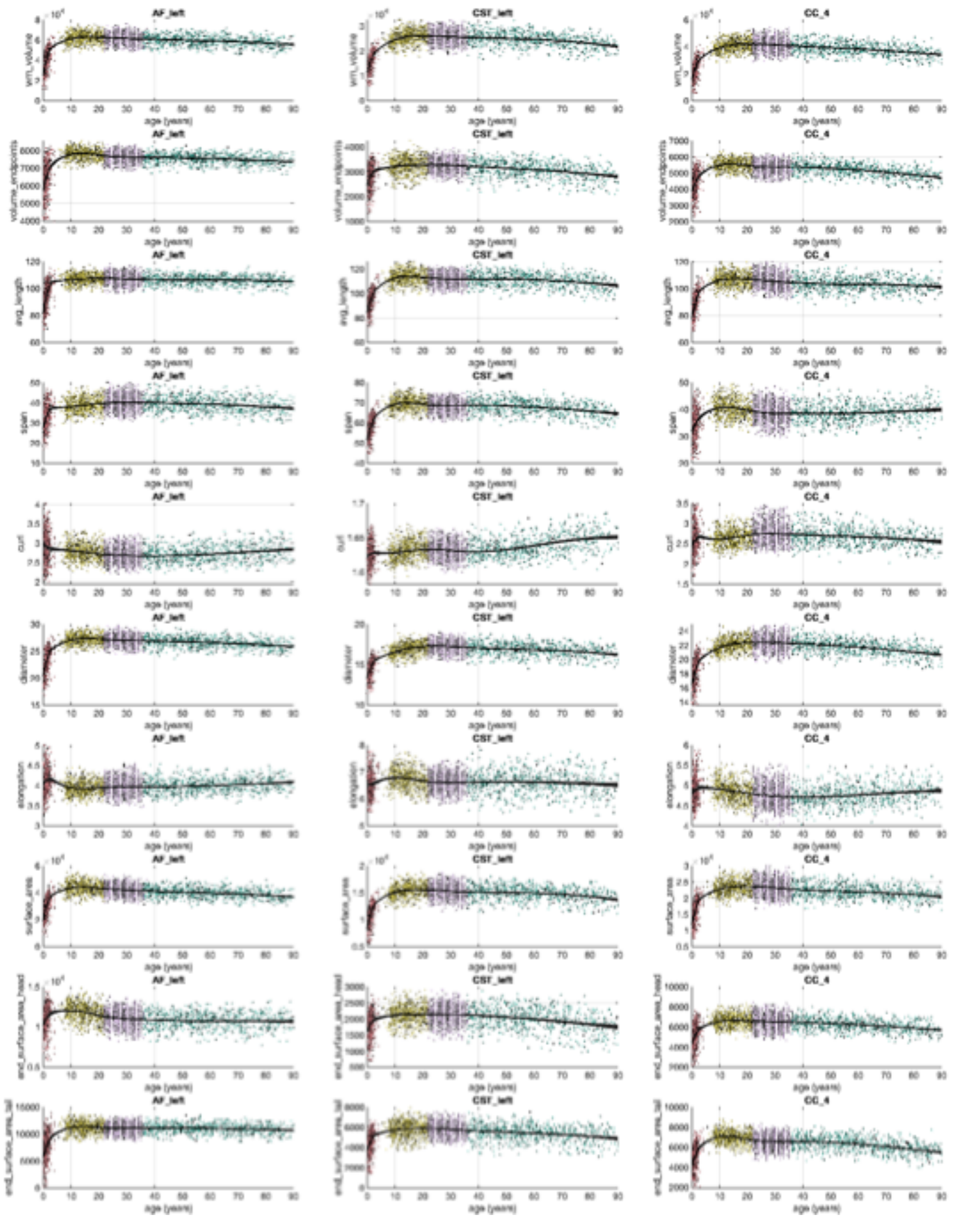

***Supplementary Figure 3**. White matter macrostructure shows unique lifespan trajectories. Lifespan trajectories of volume, volume of endpoints, average length, span, curl, diameter, elongation, surface area, and surface area of head/tail are shown for the AF_left, CST_left, and CC_4.*

Figure 6 of the main manuscript shows the lifespan trajectories of the cortical thickness associated with each white matter bundle. Trajectories of additional cortical features are given in **Supplementary Figure 4**.

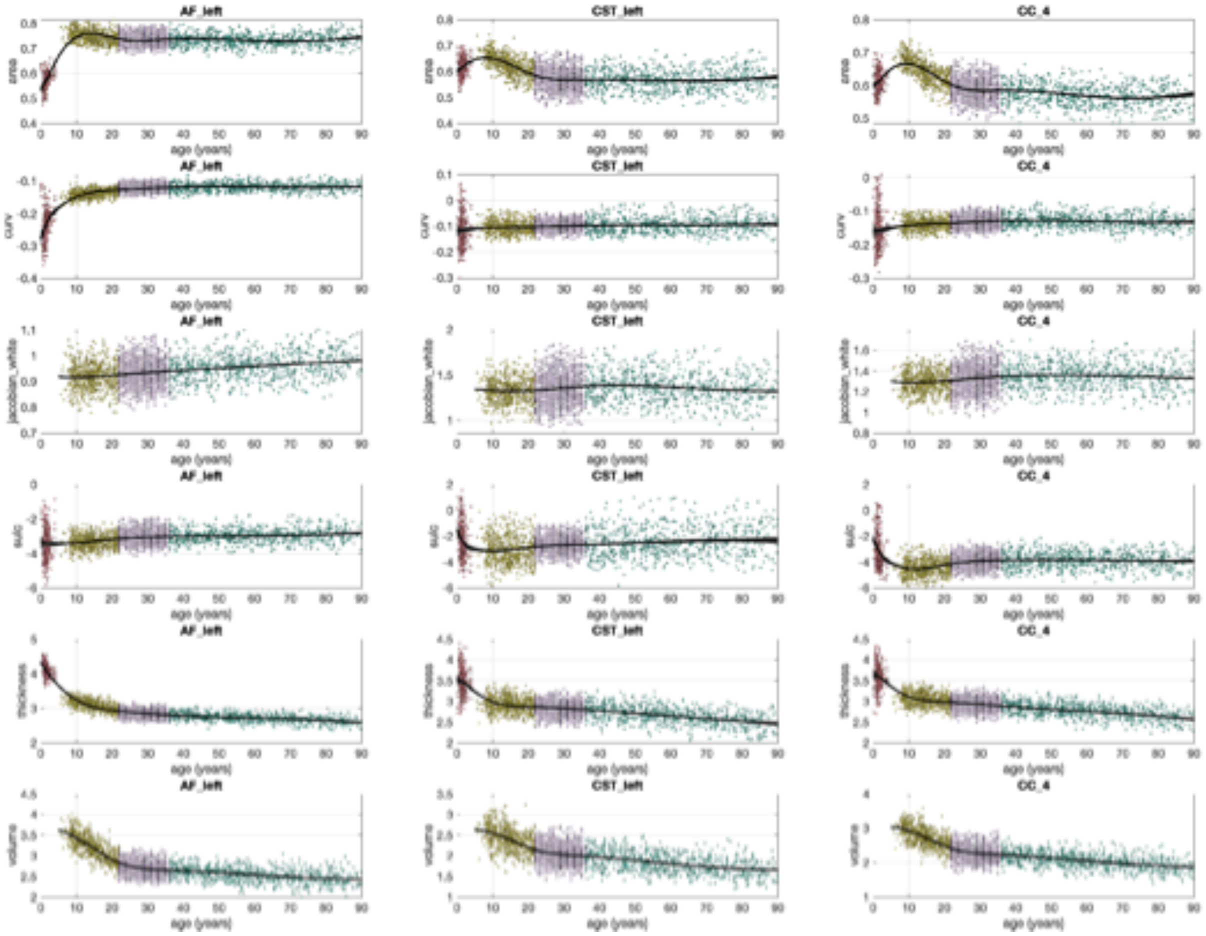

Supplementary Figure 3*. The cortical structure associated with white matter pathways shows unique lifespan trajectories. Lifespan trajectories of cortical area, curvature, jacobian of the white matter, sulcal depth, cortical thickness, and cortical volume are shown for the AF_left, CST_left, and CC_4.*

The relationship between features (correlation coefficient) at different stages of the lifespan is given in the main manuscript as **Figure 10**, for three pathways. These results averaged across all investigated pathways are shown in **Supplementary Figure 6**, and results for partial correlations in **Supplementary Figure 7**.

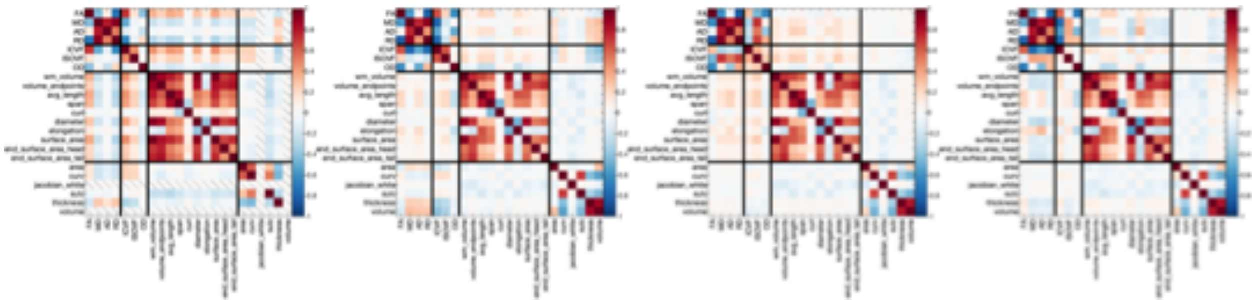

Supplementary Figure 6. Correlations exist among white matter features of microstructure, macrostructure, and cortex, that change with age. Correlation coefficients are shown between all features, averaged across all investigated pathways, for each of four cohorts (left to right).

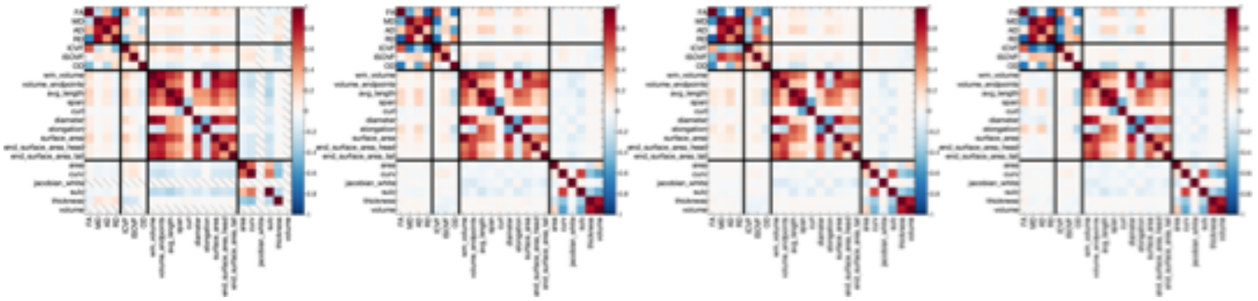

Supplementary Figure 7. Correlations exist among white matter features of microstructure, macrostructure, and cortex, that change with age. Partial correlation coefficients regressing out age are shown between all features, averaged across all investigated pathways, for each of four cohorts (left to right).

Figure 12 of the main manuscript shows several examples of features where difference per year in the Development cohort are plotted against differences per year in the Aging cohort – where selected features are shown. For completeness, **Supplementary Figure 8** shows similar results for all investigated features.

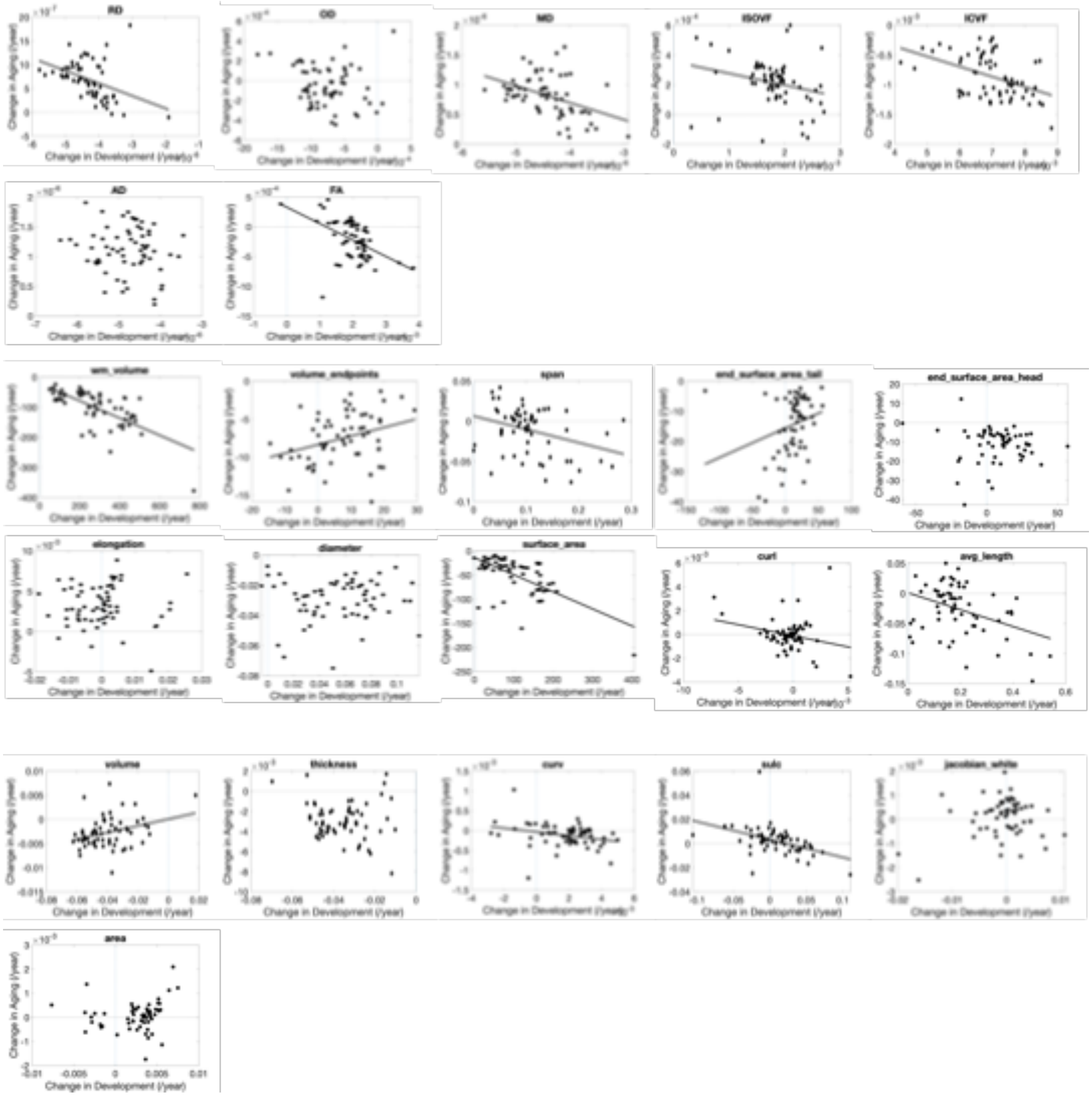

Supplementary Figure 8. *Rates of change during development are strongly related to rates of change in aging. For each plot, datapoints represent a different pathway, and statistically significant linear correlations (correcting for multiple comparisons) are shown as solid lines.*

1. Fortin, J.P., et al., *Harmonization of multi-site diffusion tensor imaging data.* Neuroimage, 2017. **161**: p. 149-170.
2. Schwarz, G., *Estimating the Dimension of a Model.* The Annals of Statistics, 1978. **6**(2): p. 461-464, 4.
